# Examining the Impacts of CO_2_ Concentration and Genetic Compatibility on Perennial Ryegrass—*Epichloë festucae* var *lolii* Interactions

**DOI:** 10.3390/jof6040360

**Published:** 2020-12-11

**Authors:** Jennifer Geddes-McAlister, Arjun Sukumaran, Aurora Patchett, Heather A. Hager, Jenna C. M. Dale, Jennifer L. Roloson, Nicholas Prudhomme, Kim Bolton, Benjamin Muselius, Jacqueline Powers, Jonathan A. Newman

**Affiliations:** 1 Department of Molecular and Cellular Biology, University of Guelph, Guelph, ON N1G 2W1, Canada; asukum02@uoguelph.ca (A.S.); nprudhom@uoguelph.ca (N.P.); bmuseliu@uoguelph.ca (B.M.); 2 Mass Spectrometry Facility—Advanced Analysis Centre, University of Guelph, Guelph, ON N1G 2W1, Canada; 3 Department of Integrative Biology, University of Guelph, Guelph, ON N1G 2W1, Canada; aurorapatchett@gmail.com (A.P.); hhager@uoguelph.ca (H.A.H.); jdale03@uoguelph.ca (J.C.M.D.); rolo1320.jr@gmail.com (J.L.R.); kbolton@uoguelph.ca (K.B.); jpower06@uoguelph.ca (J.P.)

**Keywords:** *Epichloë festucae* var. *lolii*, *Lolium perenne* L., metabolomics, quantitative proteomics, plant defense response, climate change, genetic compatibility, mutualism

## Abstract

Perennial ryegrass (*Lolium perenne*) is the most cultivated cool-season grass worldwide with crucial roles in carbon fixation, turfgrass applications, and fodder for livestock. *Lolium perenne* forms a mutualism with the strictly vertically transmitted fungal endophyte, *Epichloë festucae* var *lolii*. The fungus produces alkaloids that protect the grass from herbivory, as well as conferring protection from drought and nutrient stress. The rising concentration of atmospheric CO_2_, a proximate cause of climatic change, is known to have many direct and indirect effects on plant growth. There is keen interest in how the nature of this plant–fungal interaction will change with climate change. *Lolium perenne* is an obligately outcrossing species, meaning that the genetic profile of the host is constantly being reshuffled. Meanwhile, the fungus is asexual implying both a relatively constant genetic profile and the potential for incompatible grass–fungus pairings. In this study, we used a single cultivar, “Alto”, of *L. perenne*. Each plant was infected with one of four strains of the endophyte: AR1, AR37, NEA2, and Lp19 (the “common strain”). We outcrossed the Alto mothers with pollen from a number of individuals from different ryegrass cultivars to create more genetic diversity in the hosts. We collected seed such that we had replicate maternal half-sib families. Seed from each family was randomly allocated into the two levels of the CO_2_ treatment, 400 and 800 ppm. Elevated CO_2_ resulted in an *c*. 18% increase in plant biomass. AR37 produced higher fungal concentrations than other strains; NEA2 produced the lowest fungal concentrations. We did not find evidence of genetic incompatibility between the host plants and the fungal strains. We conducted untargeted metabolomics and quantitative proteomics to investigate the grass-fungus interactions between and within family and treatment groups. We identified a number of changes in both the proteome and metabalome. Taken together, our data set provides new understanding into the intricacy of the interaction between endophyte and host from multiple molecular levels and suggests opportunity to promote plant robustness and survivability in rising CO_2_ environmental conditions through application of bioprotective epichloid strains.

## 1. Introduction

Grasslands are important biomes throughout the world, and their responses to climatic change are the subject of much research [1]. Prominent among this work is the study of perennial ryegrass (*Lolium perenne* L.). This grass is native to Europe but widely planted and naturalized throughout the temperate world [2]. It is economically important to the dairy and beef industries [3] as well as in many turfgrass applications [4]. Perhaps because of its economic importance, there have been many studies of this plant’s response to climatic change generally and rising CO_2_ concentrations more specifically. Much of this work was done in growth chambers in the mid to late 1990s, with a few notable longer term Free Air CO_2_ Enrichment (FACE) experiments in the late 1990s and early 2000s. *Lolium perenne* responds positively to elevated CO_2_, with: increasing rates of photosynthesis, carbon assimilation, and net primary production [5,6,7,8,9]; Specific Leaf Area (SLA) declines, while Leaf Area Index (LAI) tends to increase [10,11,12,13,14,15]; and plants show faster rates of leaf and tiller elogation, higher rates of tillering and greater tiller densities [5,11,12,13,15,16,17,18,19,20,21]. Elevated CO_2_ also leads to: greater yield, greater biomass both above- and belowground, and higher root to shoot ratios of biomass allocation [5,6,8,11,15,17,21,22,23,24,25,26,27,28,29,30,31,32,33]; nitrogen (N) concentrations tend to decline [10,34], N cycling rate tends to increase while harvested N declines [35], crude protein concentrations increase while crude fibre concentrations decrease [30], soluble protein concentrations decline, as do chlorophyll concentrations, while high molecular weight carbohydrates increase [25]. It is important to note that many of these conclusions show a complex interaction with N supply.

Perennial ryegrass is often found in a mutualistic relationship with an obligate fungal endophyte *Epichloë festucae* var *lolii* (≡ *Neptyphodium lolii* ≡ *Acremonium lolii*; [36]). The *Epichloë* genus is a monophyletic clade of systemic endophytic fungi that colonize most aboveground tissues but are usually absent from the roots. Some *Epichloë* species cause “choke disease” whereby the fungus suppresses the host’s seed production, using the culms as a site to produce fungal ascospores that disperse to infect new host plants. However, many *Epichloë* are also able to colonize the grass ovary, ovule, and embryo asymptomatically without damaging the seed and transmit themselves vertically from one host generation to the next. Indeed, some *Epichloë* exhibit choke disease on some tillers and seed colonization on other tillers. Thus, these species simultaneously undergo sexual recombination combined with horizontal transmission between hosts, and clonal reproduction coupled with vertical transmission from one host generation to the next. Still other species do not reproduce sexually, and transmission to the next generation is *restricted* to vertical transmission [37]. Strictly vertical transmission means that the endophyte must evolve into a mutualist if it is to persist in the grass population (for review and discussion see [38]). However, some have challenged the notion that strictly vertically transmitted endophytes are mutualists, particularly in native grasses. For example ([39], p. 25; emphasis added),

“Endophytic fungi, especially asexual, systemic endophytes in grasses, are generally viewed as plant mutualists, mainly through the action of mycotoxins, such as alkaloids in infected grasses, which protect the host plant from herbivores. Most of the evidence for the defensive mutualism concept is derived from studies of agronomic grass cultivars, which may be atypical of many endophyte-host interactions. I argue that endophytes in native plants, even asexual, seed-borne ones, rarely act as defensive mutualists. In contrast to domesticated grasses where infection frequencies of highly toxic plants often approach 100%, natural grass populations are usually mosaics of uninfected and infected plants. The latter, however, usually vary enormously in alkaloid levels, from none to levels that may affect herbivores. *This variation may result from diverse endophyte and host genotypic combinations* that are maintained by changing selective pressures, such as competition, herbivory and abiotic factors....”

Here, and elsewhere, Faeth and colleagues posit “genetic incompatibility” between the host plant’s changing genotypes and the much more slowly changing endophytic fungus’s genotype as a mechanism that can give rise to *parasitic* vertically transmitted fungi.

The most recent taxonomic revision of this genus [36] shows that it comprises 34 species, three subspecies, and six varieties (i.e., 43 distinct lineages). Whether these species reproduce: only sexually (two species plus one subspecies), only asexually (23 species plus five varieties), or both sexually and asexually (nine species plus two subspecies) largely determines their mode(s) of transmission into the next generation. Sexual reproduction in these species provides the ability for horizontal transmission of the fungus from one individual host plant to another. Strictly asexually reproducing species seem to be limited exclusively to vertical transmission into the next generation.

*Epichloë festucae* var *lolii* is a strictly vertically transmitted species. The host–fungus relationship has been described as a “defensive mutualism” because the plant benefits from alkaloids produced by the fungus that seem to protect the plant from some forms of herbivory [40]. The fungus also improves grass performance under drought and nutrient stress [41,42,43,44], although these reported benefits are not universal (see e.g., [45] for review and further references). As a result of these benefits, endophyte infected pastures are more productive and exhibit better persistence (see e.g., [46] for review and further references). Unfortunately for farmers, the common toxic strain of the endophyte causes a condition in grazing mammals called “ryegrass staggers”. In a review article, (Cunningham and Hartley [47] p. 1) described the situation thus:

“Ryegrass Staggers is the name given to a condition of tetanic muscle spasm that develops under certain conditions in grazing sheep, cattle, or horses. In most cases, the pastures on which animals become affected have contained a considerable proportion of perennial ryegrass, and this has given rise to the name, though there is no direct proof that ryegrass is the cause; at least one out-break has occurred on short-rotation ryegrass.”

It was not until the early 1980s that researchers discovered the association of the affliction with the presence of a potent neurotoxic indole-diterpene alkaloid, lolitrem B, produced by the *Epichloë* fungi [48,49]. This tension between pasture production and persistence on the one hand, and detriments to animal health on the other, caused plant breeders to look for so called “safe endophytes.” They have attempted to capitalize on the natural variation among endophyte strains in their alkaloid production profiles, selecting different strains for different applications, and transferring these fungi to elite seed lines for commercialization [50,51]. Several promising strains have been discovered and a few have been brought to market, including AR1, AR37, and NEA2. The common toxic strain (Lp19) of the endophyte, usually denoted simply as E+, produces: lolitrem B, peramine, and ergovaline. NEA2 produces all three alkaloids but at more moderate levels than the E+ strain. AR1 produces only peramine, while AR37 does not produce any of the three alkaloids found in the E+ strain but rather produces a different set called epoxy-janthitrems (see [52] and references therein). These so-called “novel” strains induce other metabolic changes in the host plants beyond the differences in alkaloid production [53,54,55]. A similar plant breeding strategy has been taken with a closely related grass-endophyte system *Schedonorus arundinaceus* (Schreb.) Dumort. (≡ *Festuca arundinacea*; [56])–*Epichloë coenophiala* (≡ *Neotyphodium coenophialum* ≡ *Acremonium coenophialum*; see [57] and references therein).

Despite the obvious interest in the effects of climatic change on grasses (see e.g., [58]) and on the role that fungal endophytes, particularly epichloid endophytes, play in grasses and grasslands (see e.g.,  [59]), surprisingly little research has focused on the intersection of these two topics. Given the economic importance of this mutualism, it is important to ask how stable it is in the face of climatic change, particularly rising atmospheric CO_2_, which is known to produce widespread impacts on plant physiology and plant-herbivore relationships [60]. In cool-season grasses, only seven studies have investigated this topic. In the perennial ryegrass system there have only been two studies. Hunt et al. [25] investigated the impacts of a relatively small increase in CO_2_ concentration (368 vs. 466 ppm) using only the common toxic strain and endophyte-free (E−) plants. They found interactions between CO_2_ and endophyte in the plants’ production of high molecular weight and total carbohydrates as well as protein concentrations. In the E+ plants, peramine and ergovaline concentrations tended to decrease under high N conditions but only in ambient CO_2_. Marks and Clay [61] compared the performance of E− and E+ plants in 350 vs. 650 ppm CO_2_. They found that endophyte infection had little impact on plant growth except for the root:shoot ratio and that there was little indication of interactions involving endophyte and CO_2_. In the closely related *Schedonorus arundinaceus*–*Epichloë coenophiala* system, Marks and Lincoln [62] investigated the anti-herbivore properties of endophyte infection (ambient vs. 700 ppm CO_2_). The only plant-focused measure they reported was leaf N concentration, which was not affected by CO_2_, endophyte presence, or their interaction. Newman et al. [63] studied the problem in open topped chambers in the field (ambient vs. 700 ppm) and found endophyte (presence vs. absence) by CO_2_ interactions in total crude protein, soluble crude protein and acid detergent insoluble crude protein. Ryan et al. [64,65] studied higher concentrations of elevated CO_2_ (ambient, 800 and 1000 ppm) and found that fungal derived alkaloid concentrations were higher under elevated CO_2_, as were the endophyte concentrations themselves. In a field experiment, Brosi et al. [66] studied the factorial effects of a +300 ppm increase in CO_2_, a +3 °C increase in temperature, and a “dry” (2 mm H_2_O/week) or “wet” (25 mm H_2_O/week) treatment. They found higher endophyte-infection frequencies but a 30% decrease in concentrations of the alkaoilds ergovaline and loline in elevated CO_2_. While Brosi et al. Brosi et al. [66] did find changes in the concentrations of some metabolites due to elevated CO_2_, these differences seemed to be independent of endophyte presence.

In this study, we made use of untargeted metabolomic and quantitative proteomic techniques to investigate how the interactions between host plants and their associated strains of *E*. *festucae* var. *lolli* change with rising concentrations of CO_2_. These tools provide insights into the mechanisms of the relationship between the genomes of the plant and endophyte and their resulting phenotypes. These approaches are not new to the study of *Epichloë* endophytes, although they are not yet common. Untargeted metabolomics has been used to study both the *S*. *arundinaceus*–*E*. *coenophiala* system [67,68,69] and the *L*. *perenne*–*E*. *festucae* var. *lolli* system [70,71]; see Rasmussen et al. [72] for review. Similarly, a variety of proteomic approaches have been used in both systems [73,74]. See Porras-Alfaro and Bayman [75] for a general review of the usefulness of these techniques to probe plant–endophyte relationships.

In this paper we investigated several questions simultaneously:

1. Do different strains of *E. festucae* var. *lolii* produce similar fungal concentrations in a genetically diverse host plant background?
2. Do different strains of the fungus differentially moderate the impacts of elevated CO_2_ on the growth and seed production of perennial ryegrass?
3. Are the metabolomes of the host plant–fungal strain combinations different from each other and how are they altered by elevated CO_2_?
4. Are the proteomes of the host plant–fungal strain combinations different from each other and how are they altered by elevated CO_2_?
5. Does an integrated analysis of the proteome and metabolome data yield different insights than those gained from considering the proteome and metabolome separately?
6. Is there any evidence of host plant–fungal strain genetic incompatibility?

## 2. Materials and Methods

### 2.1. Plant Material, Growth Conditions, and Maternal Family Establishment

Perennial ryegrass (*L. perenne*) cv. Alto seeds infected with one of four strains of *E. festucae* var. *lolii*: AR1, AR37, NEA2, or E+ (sometimes referred to as the “wild type,” “common toxic strain,” or Lp19) were obtained from Barenbrug Agriseeds Limited (Christchurch, New Zealand; Table 1). Endophyte presence was confirmed for infected seeds by immunoblotting ten seeds from each strain prior to planting (Phytoscreen seed endophyte detection kit, Agrinostics Ltd, Co., https://www.agrinostics.com). We did not reconfirm the strain identifications. Individual seeds were grown in sterilized #4 Sunshine Mix Potting Soil (http://www.sungro.com) and watered with deionized water every other day. Additionally, perennial ryegrass seeds of other diverse cultivars (forage cultivars: Herby E− and Feeder E−, turf cultivars: Penguin E+ and Top Gun E+) were grown for cross-pollination. All plants were grown in the Edmund C. Bovey Building Greenhouse Complex at the University of Guelph (Guelph, ON, latitude 43°33${}^{\prime }$ N, longitude 80°15${}^{\prime }$ W) under approximately 40% relative humidity, 23 °C, and a light/dark 18/6 h photocycle. Flowering was induced by placing the plants at 4 °C with no light for six weeks followed by greenhouse conditions (after acclimatization), and seed heads were harvested 12 weeks later. Alto plants were fertilized by random pollen from all of the plants from all of the cultivars. Because perennial ryegrass is self-incompatible, the seeds collected from single plants represent families of half-siblings.

### 2.2. Chamber Experiment and Harvested Plant Tissue

We picked maternal families of each endophyte strain such that there were at least 24 seeds in the family. This selection yielded unequal numbers of families across the four endophyte strains (see Table 1). Seeds were sown individually in sterilized #4 Sunshine Mix Potting Soil (http://www.sungro.com) and watered and fertilized regularly with Nutricote 13-13-13 (N-P-K) with micronutrients at an application rate of 400 g/m^2^. After approximately three weeks of growth, tillers from each plant were immunoblotted to confirm endophyte infection prior to transferring the plants to pots (Phytoscreen field tiller endophyte detection kit, Agrinostics Ltd, https://www.agrinostics.com). Conviron growth chambers (Model PCG20, https://www.conviron.com) were set to long day 16/8 h, light intensity 300 µmol m^−2^ s^−1^, a constant temperature of 20 °C, and a relative humidity of 60%, with one set to 400 ppm CO_2_ (i.e., approximately the current ambient atmospheric concentration) and the other to 800 ppm CO_2_ (i.e., approximately twice the ambient concentration). Twelve plants from each family were placed in each growth chamber. Throughout the experiment, the plants and treatments were alternated weekly between the two growth chambers to try to minimize possible effects of the pseudoreplication [76]. The only exception to this procedure was for approximately two weeks during which the chambers suffered an infestation of thrips. During this period we ceased alternating the plants and treatments between chambers and treated the chambers with biocontrol mites. Seed production was induced by cold exposure (eight weeks at 6 °C, 8 h day length). Seed heads were harvested at approximately 34 weeks, air dried at room temperature for five days, and stored at −20 °C. Finally, tissue samples from full leaf blade were harvested for metabolomic analysis and pseudostem sheath harvested for endophyte quantification via qRT-PCR and for proteomics analyses. Tissue specific gene expression has also been shown in cool-season grass, with fungal genes more highly expressed in pseudostem [77]. These plant samples were flash frozen in liquid N, freeze-dried, and weighed prior to storage at −80 °C. The remaining biomass was cut at soil level, oven dried at 60 °C, and weighed.

### 2.3. Metabolomic Sample Preparation

Plant tissue (50 mg) was resuspended in 300 µL 75% cold methanol in a siliconized microcentrifuge tube and mixed on a Geno Grinder (https://www.spexsampleprep.com/2010genogrinder) for six min at 1750 rpm with one small ball bearing. Samples were centrifuged for five min at max speed with slow ramp speeds, the ball bearing was removed, and 180 µL was collected and stored as the “organic extract”. Next, 150 µL cold dH_2_O and 400 µL cold chloroform was added to the remaining sample and samples were mixed on the Gene Grinder for six min at 1750 rpm and centrifuged for 10 min at max speed. The aqueous fraction was collected and filtered through a 0.45 µm filter into a glass vial, deemed “aqueous extract”. Samples were stored at −80 °C until measured on the mass spectrometer.

### 2.4. Proteomic Sample Preparation

Plant tissue (30 mg) was processed as previously described with modifications [78]. Briefly, samples were resuspended in 100 mM Tris-HCl (pH 8.5) containing a cOmplete^™^ protease inhibitor cocktail tablet (https://www.sigmaaldrich.com). Using a probe sonicator (https://www.fishersci.ca), samples were mixed in an ice bath for 3 cycles (30% power, 30 s on/30 s off), and 2% (final) sodium dodecyl sulphate (SDS) and 10 mM dithiothreitol (DTT) was added, followed by incubation at 95 °C for 10 min with shaking at 800 rpm. The samples were cooled, and 55 mM iodoacetamide (IAA) was added, followed by incubation at room temperature for 20 min in the dark. Next, 100% ice cold acetone (final concentration of 80%) was added prior to storage at −20 °C overnight. Samples were collected by centrifugation at 13,500 rpm at 4 °C for 10 min, washed twice with 80% acetone, and air dried. Pellets were resolubilized in 8M urea/40 mM HEPES, and a bovine serum albumin (BSA) tryptophan assay determined protein concentrations [79]. Samples were diluted in 50 mM ammonium bicarbonate and digested overnight with a mixture of LysC and trypsin proteases (https://www.promega.ca, protein:enzyme ratio, 50:1). Digestion was stopped with 10% *v*/*v* trifluoroacetic acid (TFA), and 50 µg of the acidified peptides was loaded onto STop And Go Extraction (STAGE) tips (consisting of three layers of C18) to desalt and purify according to the standard protocol [80]. Samples were stored as dried peptides at −20 °C until measurement on the mass spectrometer.

### 2.5. Mass Spectrometry

For analysis of the metabolome, liquid chromatography–mass spectrometry analyses were performed on an Agilent 1200 high performance liquid chromatography (HPLC) system interfaced with an Agilent UHD 6540 Q-Tof mass spectrometer (https://www.agilent.com). The instrument was run in both positive and negative modes. A C18 column (Agilent AdvanceBio Peptide Map, 50 mm × 2.1 mm 2.7 um) was used for chromatographic separation with: A (water with 0.1% formic acid) and B (acetonitrile with 0.1% formic acid). The mobile phase gradient was: initial conditions 2% B for 2 min increased to 15% B over 13 min, followed by 50% B for 10 min. Column wash was performed at 98% B and 10 min re-equilibration. The first two and last five min of the gradient were sent to waste and not the spectrometer. The flow rate was maintained at 0.2 mL/min. The mass spectrometer electrospray capillary voltage was maintained at 4.0 kV and the drying gas temperature at 350 °C with a flow rate of 13 L/min. Nebulizer pressure was 40 psi and the fragmentor was set to 150 V. Nitrogen was used as both nebulizing and drying gas and collision-induced gas. The mass-to-charge (*m/z*) ratio was scanned across the *m/z* range of 200–2000 in 4 GHz (extended dynamic range positive-ion auto MS/MS mode). Three precursor ions per cycle were selected for fragmentation. The instrument was externally calibrated with the ESI TuneMix (https://www.agilent.com). The sample injection volume was 20 µL. Triplicate technical replicates were performed for all biological replicates.

For analysis of the proteome, samples were eluted from STAGE-tips with 50 µL buffer B (80% acetonitrile (ACN) and 0.5% acetic acid), dried, and resuspended in 12 µL buffer A (0.1% TFA). Six µL of each sample was analyzed by nanoflow liquid chromatography on an Ultimate 3000 LC system (https://www.thermofisher.com) online coupled to a Fusion Lumos Tribrid mass spectrometer (https://www.thermofisher.com) through a nanoelectrospray flex-ion source (https://www.thermofisher.com). Samples were loaded onto a 5 mm µ-precolumn (https://www.fishersci.ca) with 300 µm inner diameter filled with 5 µm C18 PepMap100 beads. Peptides were separated on a 15 cm column with 75 µm inner diameter with 2 µm reverse-phase silica beads and directly electrosprayed into the mass spectrometer using a linear gradient from 4% to 30% ACN in 0.1% formic acid over 45 min at a constant flow of 300 nL/min. The linear gradient was followed by a washout with up to 95% ACN to clean the column followed by an equilibration stage to prepare the column for the next run. The Fusion Lumos was operated in data-dependent mode, switching automatically between one fill scan and subsequent MS/MS scans of the most abundant peaks with a cycle time of 3 s. Full scan MS1s were acquired in the Orbitrap analyzer with a resolution of 120,000, scan range of 400–1600 *m/z*. The maximum injection time was set to 50 ms with an automatic gain control target of $4\times 10^{5}$. The fragment ion scan was done in the Orbitrap using a Quadrupole isolation window of 1.6 *m/z* and HCD fragmentation energy of 30 eV. Orbitrap resolution was set to 30,000 with a maximum ion injection time of 50 ms and an automatic gain control target set to $5\times 10^{4}$.

### 2.6. Omics Data Analysis

For metabolome data analysis, spectra processing was performed using Batch Recursive Feature Extraction in Mass-hunter Profinder version B.08.00 (https://www.agilent.com). Profinder recursive feature extraction involves an initial naïve feature finding algorithm, Molecular Feature Extraction (MFE), which combines coeluting related ions such as adducts or different charge states into one compound. This list of compounds is then verified in a second round of feature finding with the Find by Formula algorithm, which uses the ion *m/z* values and isotope ratios found by MFE to reinterrogate the data. The initial *m/z* threshold set for feature detection was 300 counts and extraction window of 40 ppm using the Molecular Feature Extraction algorithm. After isotope grouping using the peptide isotope model, the compound threshold was set to 3000 counts in at least two-thirds of samples in one group. For the recursive portion of the feature detection, a list of consensus metabolites determined from all samples was used to reassess the raw data using the Find by Ion algorithm using a 50-ppm extracted ion chromatograph (EIC) extraction window. Defined masses were searched against Formula, Metlin AM, Metlin Metabolites, and in-house KnapSack for compound identification. The data were exported from Profinder to Perseus (version 1.6.2.2; [81]), and intensities were log_2_-transformed and classified according to groups (endophyte strain, maternal family, CO_2_ levels). Values were filtered based on valid values (metabolite identification required in two of three replicates in at least one group), followed by imputation based on the normal distribution. Statistical processing included a Student’s *t*-test for identification of metabolites with large changes in abundance among samples (p≤0.05, s≥4.32), with multiple hypothesis testing correction using the Benjamini–Hochberg False Discovery Rate (FDR) [82] cutoff at 0.05. The mass spectrometry metabolomics data are available upon request from the corresponding authors.

For proteome data analysis, *.Raw files were analyzed using MaxQuant software (version 1.6.0.26.) [83]. The derived peak list was searched with the built-in Andromeda search engine against the reference *L. perenne* (21 February 2019; 11,123 sequences; http://pgsb.helmholtz-muenchen.de/plant/index.jsp) and *E. festucae* var. *lolii* (21 February 2019; 9298 sequences; http://csbio-l.csr.uky.edu/endophyte/). The parameters were as follows: strict trypsin specificity, allowing up to two missed cleavages, minimum peptide length of seven amino acids, carbamidomethylation of cysteine as a fixed modification, N-acetylation of proteins and oxidation of methionine set as variable modifications. A minimum of two peptides required for protein identification and peptide spectral matches and protein identifications were filtered using a target-decoy approach at a FDR of 1%. “Match between runs” was enabled with a match time window of 0.7 min and an alignment time window of 20 min. Relative, label-free quantification (LFQ) of proteins used the MaxLFQ algorithm integrated into MaxQuant using a minimum ratio count of one [84]. The mass spectrometry proteomics data have been deposited in the PRIDE partner repository for the ProteomeXchange Consortium with the data set identifier: PXD017961.

Further analysis of the MaxQuant-processed data (proteingroups.txt file) was performed using Perseus (version 1.6.2.2, [81]). Hits to the reverse database, contaminants, and proteins only identified with modified peptides were eliminated. LFQ intensities were converted to a log scale (log_2_), and only those proteins present in triplicate within at least one sample set were used for further statistical processing (valid-value filter of three in five replicates in at least one group). Missing values were imputed from a normal distribution (downshift of 1.8 standard deviations and a width of 0.3 standard deviations). A Student’s *t*-test identified proteins with important changes in abundance (p≤0.05) with multiple hypothesis testing correction using the Benjamini–Hochberg FDR [82] cutoff at 0.05. A principal component analysis (PCA) was performed, as well as Pearson correlation with hierarchical clustering by Euclidean distance to determine replicate reproducibility and clustering of samples.

### 2.7. Endophyte Quantification

Endophyte infection was measured using qRT-PCR to amplify the translation elongation factor 1−α ([85], GenBank Acc. # JX028264) following Ryan et al. [64]. Genomic DNA (gDNA) was extracted from 20 mg of sheath tissue using the DNeasy Plant Mini Kit (https://www.qiagen.com), and total gDNA (plant and fungal) was determined by spectroscopy using a NanoDrop 2000 (https://www.thermofisher.com). PCR reactions were performed and analyzed on a LightCycler 480 Instrument II (https://lifescience.roche.com) using gene-specific primers (forward: 5${}^{\prime }$-cacgtactgactgaagcgtagc-3${}^{\prime }$; reverse: 5${}^{\prime }$-caatgcagcgagtgaacatc-3${}^{\prime }$). The concentration of endophyte is expressed as the number of copies of the fungal-specific gene ng^−1^ total gDNA. All reactions were performed in three biological replicates and three technical replicates.

## 3. Results

We follow Wasserstein et al. [86] in reporting exact *p*-values (where practical) and avoiding the use of the terms “significant” and “non-significant.” Furthermore, we follow Greenland [87] by also reporting the Shannon information transformation, $s=-\log_{2}\left(P\right)$. As Greenland notes, larger values of *s* correspond to more evidence against the null hypothesis. The Shannon information transformation can be interpreted as:

“This measures the amount of information supplied by the test against the tested hypothesis (or model): Rounded off, the *s*-value *s* shows the number of heads in a row one would need to see when tossing a coin to get the same amount of information against the tosses being “fair” (independent with “heads” probability of 12) instead of being loaded for heads. For example, if p=0.03, this represents $-\log_{2}\left(0.03\right)=5$ bits of information against the hypothesis (like getting 5 heads in a trial of “fairness” with 5 coin tosses); and if p=0.25, this represents only $-\log_{2}\left(0.25\right)=2$ bits of information against the hypothesis (like getting 2 heads in a trial of “fairness” with only 2 coin tosses).”

([86], p. 12)

### 3.1. Plant and Fungal Growth Responses

For this analysis, all 288 plants were used. We analyzed these data in a general linear model with plants nested in family and endophyte strain, and endophyte strain and family cross-factored with CO_2_ treatment (Plant_(24)_(Family_(2–4)_(Endophyte_4_)[CO_2_]_(2)_)). We treated Family as a random effect and used restricted maximum likelihood (REML) estimation.

**Figure 1:**
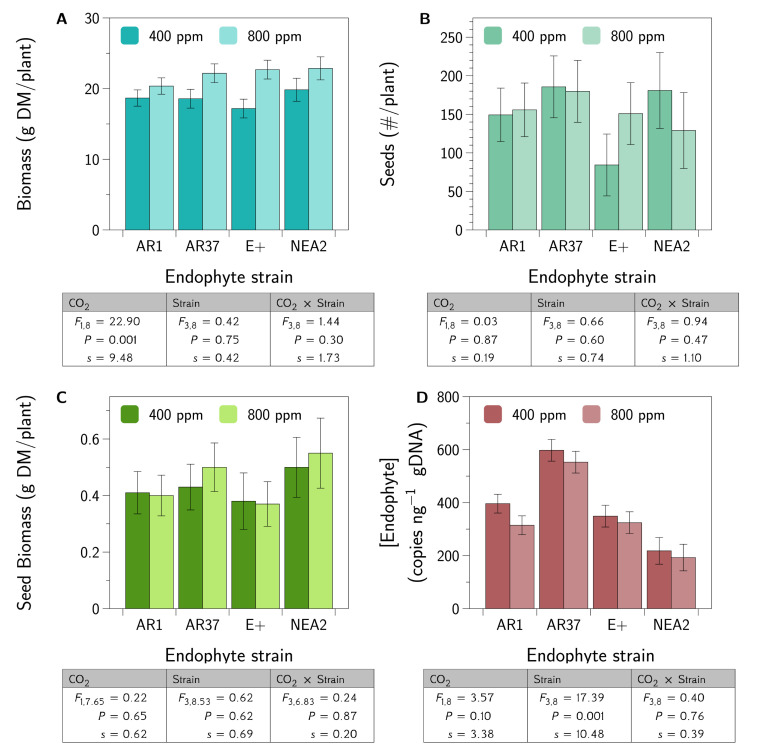
Plant growth responses—fixed effects. Shown are the fixed effects (LS Means ± SEM) from a REML analysis of the model Plant_24_(Family_2–4_(Strain_4_)[CO_2_]_2_). Below each graph are the results from the corresponding ANOVA. We also report the Shannon information transformation, *s*. (**A**) shows the mean plant biomass (g/plant). (**B**) shows the seed number per plant. (**C**) shows the seed biomass (g DM/plant). (**D**) shows the endophyte concentration (gene copies [ng^−1^ gDNA]).

The results for the fixed effects are shown in Figure 1. The main results of interest are the *c.* 18% increase in plant biomass under elevated CO_2_ and the differences among the endophyte strains in terms of the endophyte concentrations within the plants. There was no strong evidence to reject the null hypotheses for the number of seeds produced or the seed biomass.

**Figure 2:**
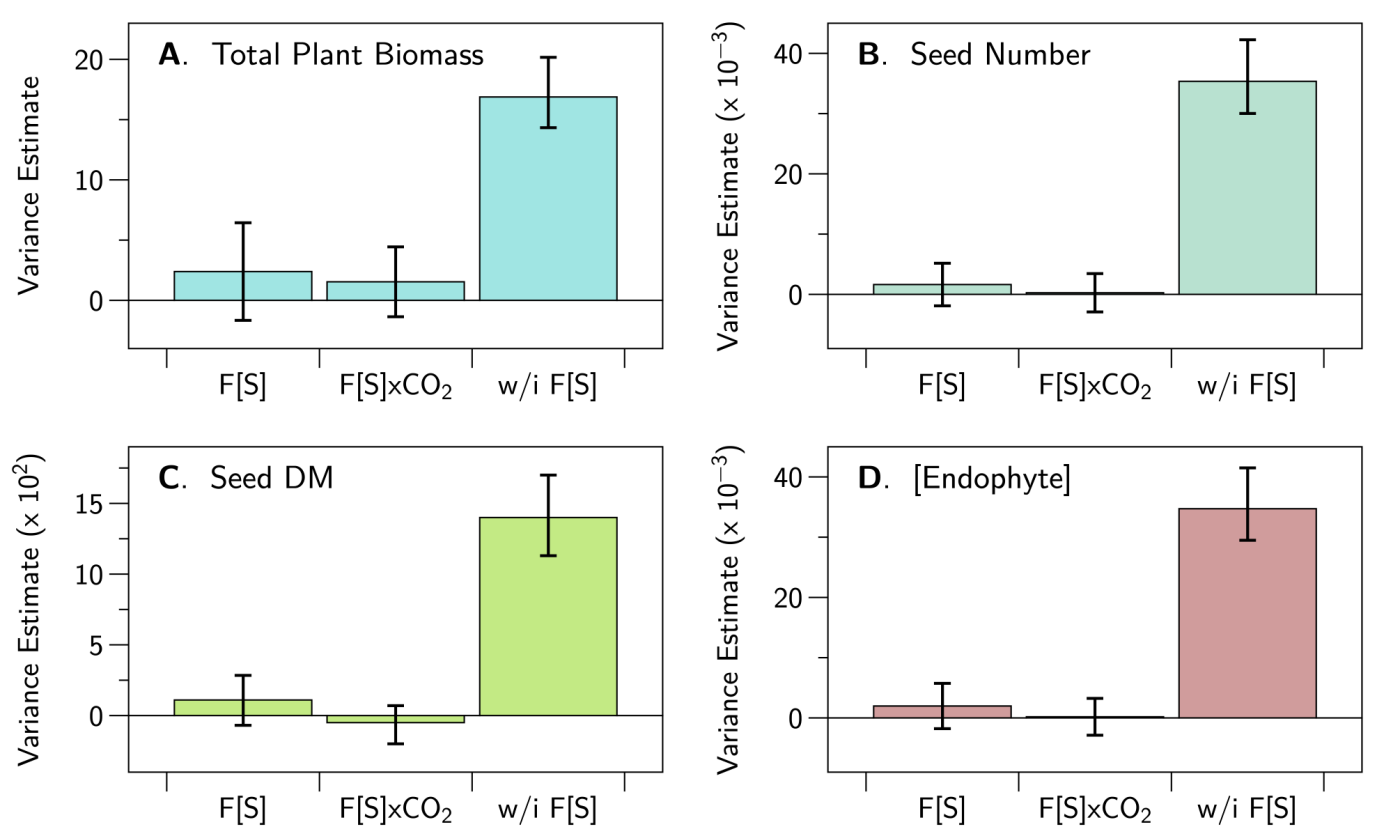
Plant growth responses—random effects. Shown are the variance estimates for the random effects. “F[S]” denotes the variance *between* maternal families nested in endophyte strain. ‘w/i F[S]’ denotes the variance *within* maternal families nested in strain. Variation between families is considerably less than variation within families, indicating no evidence of genetic incompatibility between host plants and endophyte strains. The units for the variances are: (**A**) ${\left(g\;\mathrm{DM}/\mathrm{plant}\right)}^{2}$, (**B**) ${\left(\#/\mathrm{plant}\right)}^{2}$, (**C**) ${\left(g\;\mathrm{DM}/\mathrm{plant}\right)}^{2}$, (**D**) ${\left(\mathrm{copies}/\mathrm{ng}\;\mathrm{gDNA}\right)}^{2}$.

The results for the random effects are shown in Figure 2. Briefly, there was no indication that the between family variation was larger than the within family variation, suggesting that there is no evidence that the recombination procedure we used created genetic incompatibility between the host and the endophyte.

### 3.2. Multi-OMICs Workflow

**Figure 3:**
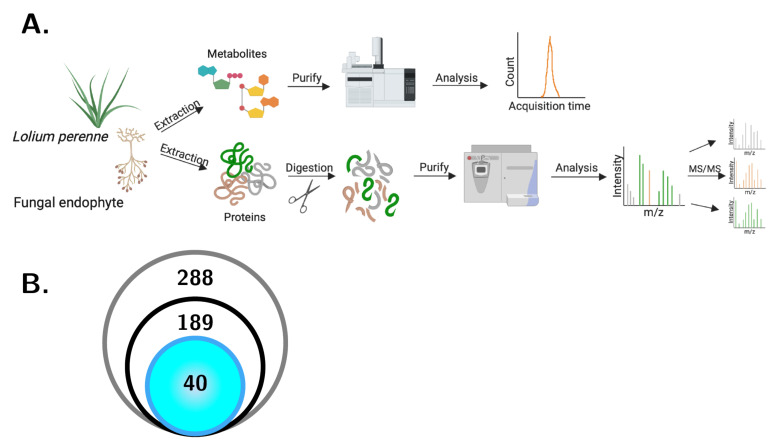
Overview of OMICs analysis. (**A**) Total plant biomass of *L. perenne* samples colonized with an endophyte (AR1, AR37, E+, or NEA2) were collected and subjected to a metabolite extraction protocol followed by mass spectrometry (LC-MS). In addition, pseudostem tissue of *L. perenne* samples colonized with an endophyte (AR1, AR37, E+, or NEA2) was collected and subjected to a protein extraction protocol followed by enzymatic digestion and tandem mass spectrometry (LC-MS/MS; figure generated using https://biorender.com). (**B**) In total, 288 samples were collected and processed for metabolomic profiling (grey). Of these, 189 samples were of substantial quality to proceed to mass spectrometry for metabolite identification (black), and a subset of 40 samples was selected for proteomic profiling (blue shading; 10 each from one family nested in each strain, of which 5 were from 400 ppm and and 5 were from 800 ppm CO_2_—see Table 1).

To assess the impact of the endophyte on *L. perenne* under altered CO_2_ conditions, we designed our experiments to profile the metabolome and proteome of the samples, followed by integration of the data set (Figure 3A). In total, 288 samples were prepared for metabolome profiling, of which, 189 were selected for analysis based on sample quality (Figure 3B). Of these, 40 samples were selected for in-depth quantitative proteomics profiling based on consistency and reproducibility of metabolite production in the metabolomics profiling.

### 3.3. Metabolic Profiling Defines Endophyte-Specific Responses

To profile changes in metabolite production among the endophytes and altered CO_2_ levels, we measured the metabolome using mass spectrometry in both positive and negative ion modes. In positive ion mode, we identified 1531 metabolites, and in negative ion mode we identified 641 metabolites.

**Figure 4:**
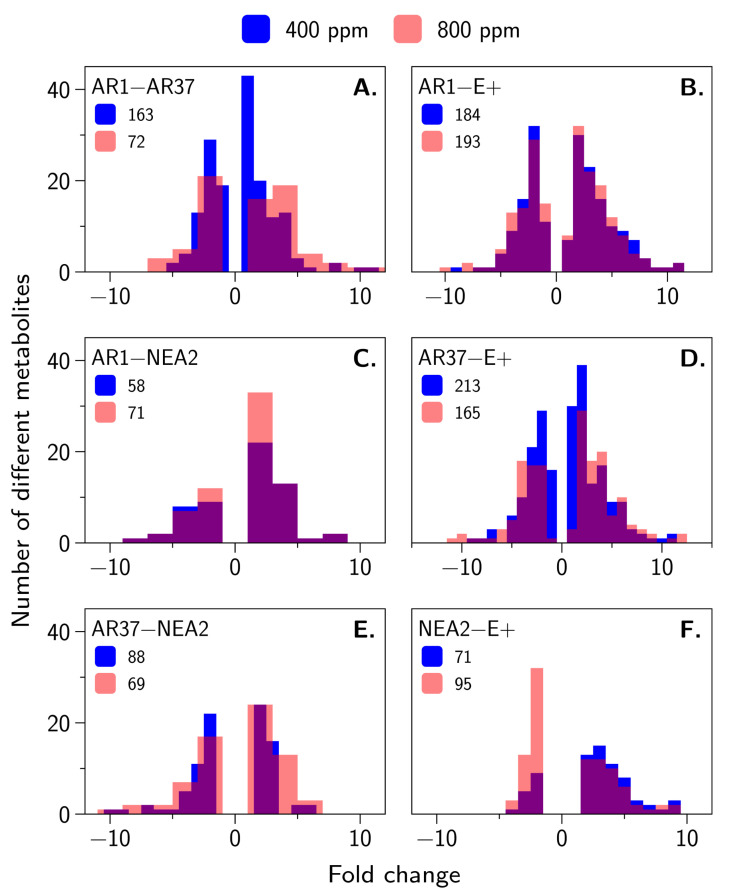
Distribution of magnitude of metabolite abundance differences. Shown are the distributions of the magnitudes of metabolite abundance differences between different endophyte strains at ambient CO_2_ (blue bars) and elevated CO_2_ (pink bars). Areas where the two distributions overlap is shown in purple. The numbers in the legends indicate the total number of metabolite differences for that comparison. (**A**) denotes the fold change differences of AR1−AR37; (**B**) denotes the fold change differences of AR1−E+; (**C**) denotes the fold change differences of AR1−NEA2; (**D**) denotes the fold change differences of AR37−E+; (**E**) denotes the fold change differences of AR37−NEA2; and (**F**) denotes the fold change differences of NEA2−E+.

Based on changes in metabolite abundance by area under the curve (Student’s *t*-test, p<0.05; FDR=0.05), we explored the relationship among 373 metabolites and the treatment effects. Of the 373 metabolites, 56 could be tentatively identified by mass and retention time based on compound mapping through MetLin, Formula, and an in-house database (see Supplemental Tables S1 and S2 for complete details). Figure 4 shows the distribution of fold-change differences for those metabolites that differed in abundance between strains. Metabolites of AR37 and AR1 are quite different from E+, and from each other, although the latter difference is reduced at elevated CO_2_. The smallest differences are between NEA2 and any of the three remaining strains. There were only two metabolites that showed large changes within a strain between elevated and ambient CO_2_. We could not identify these metabolites by name. A metabolite with an average mass of 369.1975 Da and an average retention time of 0.92 min had approximately 3-fold greater abundance in elevated CO_2_ for AR37 and E+ infected plants. A metabolite with an average mass of 238.1041 Da and an average retention time of 0.89 min was approximately 2.5-fold greater in abundance under elevated CO_2_.

**Figure 5:**
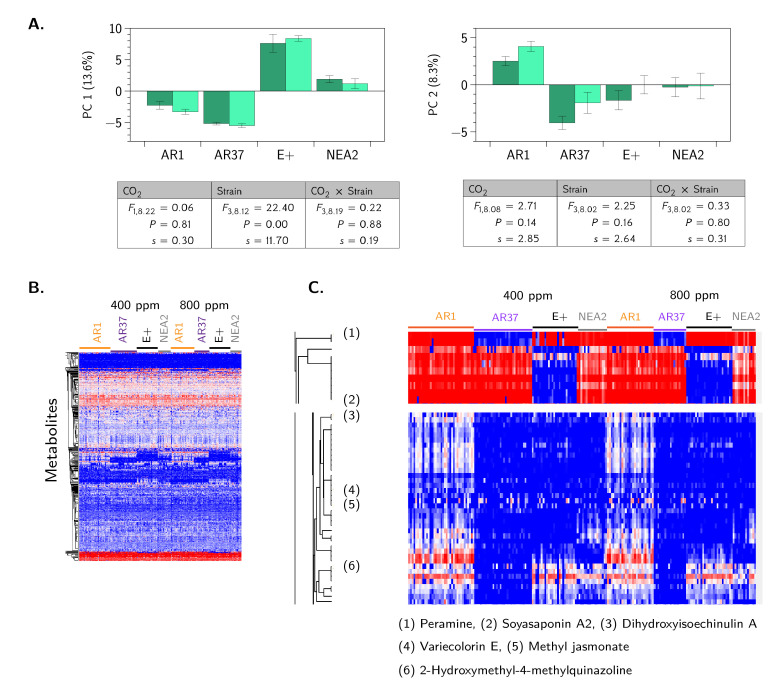
Metabolomic profiling of interaction between *L. perenne* and epichloid strains. (**A**) Principal component analysis of *L. perenne* colonized with epichloid strains (e.g., AR1, AR37, E+, NEA2); dark bars denote 400 ppm CO_2_, light bars denote 800 ppm CO_2_. “PC 1” and “PC 2” denote the first and second principal components. The ANOVA tables show the results of a REML analysis for the fixed effects of strain, CO_2_ and their interaction; random effects not shown. We also report the Shannon information transformation, *s*. Bars denote the means and standard errors. (**B**) Heat map of hierarchical clustering by Euclidean distance of all metabolites identified from *L. perenne* colonized with epichloid strains (e.g., AR1, AR37, E+, NEA2). (**C**) Regions of unique metabolite profiles highlighted and enlarged, along with the available corresponding compound identifications.

Given this coverage of changes in the metabolome, we performed a principal component analysis (PCA), which exhibited separation in samples based on *Epichloë* strain but not CO_2_ (Figure 5A). The components are well defined; they do not overlap in “heavily loading” metabolites (defined here as |x|≥0.70). These metabolites are shown in Appendix A, Table A1. Hierarchical clustering by Euclidean distance produced a heatmap demonstrating clustering of the metabolites by endophyte but not by CO_2_ levels (Figure 5B). Upon closer analysis, nine compounds showed distinct patterns of production across the tested parameters. As expected, we observed consistent differences in the production of peramine, driven largely by the multiple maternal host families of AR37, because AR37 does not produce peramine [55] (Figure 5C). A compound, tentatively identified as Soyasaponin A2, a triterpenoid glycoside traditionally identified in soybeans, was clustered with six other related but unidentifiable compounds. These metabolites showed consistent differences across endophyte strain, being largely absent from plants infected with the E+ strain. Overall, profiling of the metabolome highlights clear distinctions among the endophytes but surprisingly few large differences with changing CO_2_ levels. Future work using LC-MS/MS profiling will increase identification rates.

### 3.4. Proteomic Profiling Reveals Fungal Strain by CO_2_ Interactions

**Figure 6:**
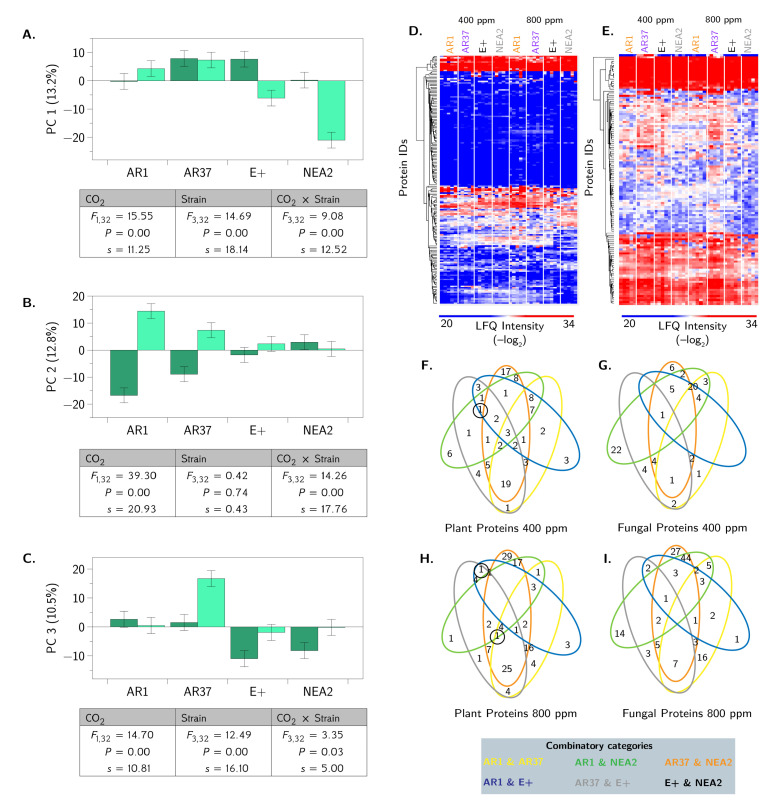
Quantitative proteomics profiling of endophyte-specific response to colonization of *L. perenne* with epichloid strains. (**A**–**C**) Principal components analysis of *L. perenne* colonized with epichloid strains (AR1, AR37, E+, NEA2); dark bars denote 400 ppm CO_2_, light bars denote 800 ppm CO_2_. Shown are the means and standard errors. (**D**) Heat map of hierarchical clustering by Euclidean distance for plant proteins that differed among the epichloid strains and at normal (400 ppm) and elevated (800 ppm) CO_2_ levels. (**E**) Heat map of hierarchical clustering by Euclidean distance for fungal proteins that differed among the epichloid strains and at normal (400 ppm) and elevated (800 ppm) CO_2_ levels. (**F**,**G**) Venn diagrams of common and unique differences in plant and fungal proteins at 400 ppm CO_2_. (**H**,**I**) Venn diagrams of the number of common and unique differences in plant and fungal proteins at 800 ppm CO_2_. The numbers in F–I indicate the number of proteins with large differences in abundance, as evaluated by Student’s *t*-tests, p<0.05, FDR=0.05, S0=1. Below each graph are the results from the corresponding ANOVA. We also report the Shannon information transformation, *s*.

We selected a single maternal family from each endophyte strain, based on consistency and reproducibility of metabolite production, for proteomic profiling (see Table 1). Our mass spectrometry-based proteomics workflow enables detection of protein-level changes from both the host (*L. perenne*) and endophyte perspectives in a single experiment. In total, we identified 890 proteins, 713 proteins belonging to *L. perenne* and 177 proteins belonging to *E. festucae* var *lolii*. Biological replicate reproducibility was >90% for all treatments (Figure S1). To better understand this multidimensional response, we performed a PCA using all 890 proteins. The first three principal components were retained for further analysis. The proteins that loaded heavily on each axis are presented in Appendix B, Table A2. These 63 proteins (16 fungal and 47 plant) account for 36.5% (13.2%, 12.8%, and 10.5%) of all the protein abundance variation. Figure 6A–C shows separation of the fungal strains by CO_2_ concentrations. For example, AR1 shows large separation by CO_2_ concentrations on PC2 but not PC3, while NEA2 segregates by CO_2_ concentrations on PC1 but not PC2 or PC3. Next we examined the univariate responses of individual proteins that differed between CO_2_ concentrations for at least one endophyte strain. Hierarchical clustering by Euclidean distance produced a heatmap demonstrating variability in protein abundance associated with CO_2_ conditions and endophyte for the plant-derived proteins Figure 6D as well as the endophyte derived proteins Figure 6E.

We performed FDR-corrected Student’s *t*-test comparisons of the differences in protein abundances for each fungal strain at 400 and 800 ppm CO_2_. We found a total of 133 different unique proteins, including 98 plant proteins and 35 fungal proteins (Figure 6 and Appendix B: Table A3 and Table A4). Of the 35 fungal proteins that changed abundances between ambient and elevated CO_2_, 27 changed only in a single endophyte strain, 14 of these in AR1. The remaining eight fungal proteins changed in exactly two strains each. Of these, five were changed in both AR1 and NEA2, but all five changed in opposite directions (increased abundance in elevated CO_2_ for AR1, and decreased abundanace for NEA2). The remaining three were changed in both AR1 and AR37 (see Appendix B, Table A3). For the 98 plant proteins, 77 were altered in only a single strain, 52 of these were only in AR1. Of the remaining 21, 13 were changed in AR1 and NEA2, and like the fungal proteins, all 13 of these changes were in the opposite directions between the strains (increase protein abundances in AR1, decreased in NEA2 under elevated CO_2_). Six more show changes in both AR1 and AR37, five of which were in the same direction (increase abundances for both strains under elevated CO_2_). There was one protein that changed in AR1, AR37, and NEA2. Consistent with the trends just mentioned, these changes were again in the same direction (increased abundances) for AR1 and AR37 and opposite directions for AR1 and NEA2 (see Appendix B, Table A4).

More generally, we examined overlap by constructing Venn diagrams using only the differently abundant proteins among all comparisons from both the plant and endophyte perspectives (Figure 6F–I). Here, considering plant protein abundance at both CO_2_ levels, we consistently observed the most common responses in the AR37 and NEA2 comparison, as well as several combinatory categories showing unique responses with identification of only one protein (e.g., AR1 and NEA2 and AR37 and E+). Conversely, for endophyte proteins, we observed variation in the number of proteins within a combinatory category. For example, at 400 ppm CO_2_, the combinatory category of AR1 and NEA2 involved the most proteins, whereas at 800 ppm CO_2_, the largest number of different proteins was found in the categories of AR1 and NEA2 combined with AR37 and NEA2. Taken together, this data set distinguishes differences in protein abundance among the endophytes and demonstrates differences in plant response in the presence of specific endophytes. Furthermore, we identified proteins commonly produced during the interaction between endophyte and host, as well as uncovered changes in protein abundance unique to specific interactions.

**Figure 7:**
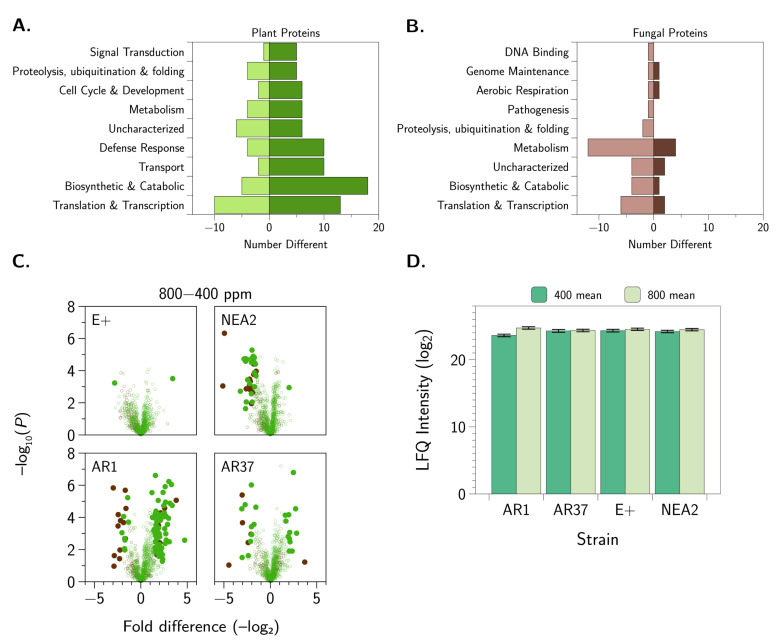
Quantitative proteomics profiling of CO_2_-mediated responses. (**A**) Distribution of differences in plant proteins based on Gene Ontology Biological processes displaying increased (dark green) or decreased (light green) abundance at 800 ppm CO_2_ levels. (**B**) Distribution of differences in endophyte proteins based on Gene Ontology Biological processes displaying increased (dark red) or decreased (light red) abundance at 800 ppm CO_2_ levels. (**C**) Volcano plots of *L. perenne* colonized with each epichloid strain at 800 vs. 400 ppm of CO_2_. Plant proteins that differed (Student’s *t*-test, p<0.05, FDR=0.05, S0=1) between the CO_2_ conditions are denoted with solid green symbols and fungal proteins that differed are denoted with solid brown symbols. Negative fold differences denote proteins that declined in abundance under elevated CO_2_, positive fold differences denote proteins that increased in abundance under elevated CO_2_. (**D**) LFQ intensity plot (mean ± standard error) of defense-related differences in plant proteins for *L. perenne* colonized with each epichloid strain (N=5). Quantification of five biological replicates. Error bars represent standard error of the mean.

Next, we aimed to define the impact of CO_2_ levels on the interaction between endophyte and host by comparing changes in protein abundance. Notably, we observed a decrease in abundance of seven endophyte proteins associated with metabolism for AR1 at elevated CO_2_. For AR37, we observed a change for 30 proteins, including one endophyte and 12 plant proteins with increased abundance at 800 ppm (Figure 7C). Colonization with E+ altered abundance of two plant proteins, including one protein with increased abundance; no endophyte proteins were different (Figure 7C). For NEA2, 39 proteins were different, including 23 plants proteins and 15 endophyte proteins with decreased abundance at elevated CO_2_ levels (Figure 7C). For endophyte proteins of NEA2, we observed increased production of a pathogenesis-associated vesicle transport protein at ambient CO_2_ levels and greater than 30-fold increases in abundance for an isomerase involved in protein folding and an uncharacterized methoxylase. The proteins that differed in each comparison (i.e., *L. perenne* colonized with AR1, AR37, E+, or NEA2 at 400 and 800 ppm CO_2_) are provided for endophyte (Appendix B, Table A3) and plant (Appendix B, Table A4) profiling. This approach enables us to identify proteins from both perspectives (plant and endophyte) with altered production influenced by epichloid strain under rising CO_2_ conditions.

To provide functional insight into the proteins with changes in abundance, we classified the plant and endophyte proteins by Gene Ontology Biological processes. For plant proteins displaying changes in abundance, the proteins fall into nine categories, with the majority of proteins associated with translation and RNA processing, biosynthetic and catabolic processes, transport, and defense response (Figure 7A). Profiling of endophyte proteins also identified nine categories with most proteins associated with metabolism, biosynthetic and catabolic processes, and translation and transcription (Figure 7B). Given our hypothesis that endophyte strains differentially influence plant responses and our observation of opposite patterns of defense protein production upon rising CO_2_ levels, we profiled changes in abundance of these proteins among the comparisons. We observed increases in plant defense-associated proteins (N=14) in the presence of AR1 at elevated CO_2_ levels, whereas each of the other endophytes showed similar or slightly increased production of defense related proteins with rising CO_2_ levels (Figure 7D). Overall, our approach promotes detection of specific plant and endophyte proteins influenced by rising CO_2_ conditions, as well as provides functional insight into the impacts of environmental change from both the plant and endophyte perspectives. Furthermore, we distinguish differences in protective properties among the epichloid strains influenced by rising CO_2_ levels, which suggests a connection among plant biomass production, protein abundance, and plant defense.

### 3.5. Integrated OMICS Reveals Important Additonal Metabolites and Proteins

**Figure 8:**
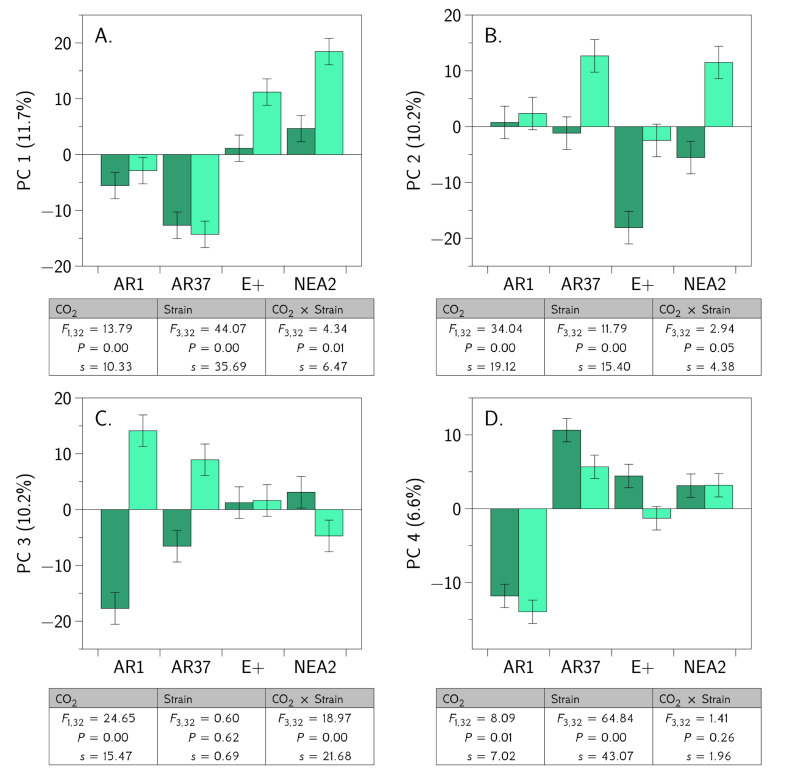
Integrated OMICs responses to CO_2_ and endophyte strain. Throughout, the dark bars represent 400 ppm CO_2_, while the light bars denote 800 ppm CO_2_. Error bars depict the standard error of the mean. (**A**) principal component 1. (**B**) principal component 2. (**C**) principal component 3. (**D**) principal component 4. Below each graph are the results from the corresponding ANOVA. We also report the Shannon information transformation, *s*.

For the subset of data for which we had proteomic data, we combined this with the corresponding metabolomics data and the corresponding estimates of the *Epichloë* concentrations and conducted a PCA. We retained the first four principal components for further analysis. We subjected these principal components to a two-way ANOVA. The results are shown in Figure 8 and the heavily loading proteins and metabolites are shown in Table A5. For the first three principal components, there was a CO_2_ × endophyte strain interaction. For the fourth principal component there was no interaction, but both main effects were important. The three interactions have varied causes. For PC-1, the interaction seems to be driven by a differential CO_2_ effect on E+ and NEA2 infected plants, while AR1 and AR37 infected plants did not respond to CO_2_ for these proteins and metabolites. For PC-2, the interaction is driven by AR37, E+ and NEA2 infected plants increasing in PC-2 under elevated CO_2_ while AR1 plants were largely unresponsive. Finally, for PC-3, the interaction is driven largely by the responses of AR1 and AR37 infected plants. The principal components are each derived from between 5 and 20 metabolites and/or proteins that change in concert with each other (although not always in the same direction; see loading signs in Appendix C, Table A5). The integrated analysis revealed four metabolites, nine fungal derived proteins, and 21 plant derived proteins that were not identified in the previous analyses, indicating the value of combining the metabolomic and proteomic data sets. These results suggest that the effects of the *Epichloë* endophytes are widespread and that the influence of CO_2_ on the plant-fungal interaction is complex.

## 4. Discussion

We begin by reexamining our original questions in light of the evidence we obtained from this experiment.

### 4.1. Do Different Strains of *Epichloë* *festucae* var. *lolii* Produce Similar Fungal Concentrations in a Genetically Diverse Host Plant Background?

We found that AR37 produced higher concentrations of the endophyte than any of the other three strains (Figure 1). In a previous study using AR1, AR37, and E+ in different perennial ryegrass cultivars (Fennema and AberDove) than used here (Alto), Rasmussen et al. [54] found that E+ produced higher concentrations than AR1, and AR1 produced higher concentrations than AR37, which is opposite to what we found. These opposing results suggest that some plant–fungal geneotype combinations may be more compatible than others in terms of fungal growth—although it is difficult to say whether endophyte concentrations are indicative of endophyte fitness. In any case, these conflicting results suggest complex host–endophyte interactions that require more experimental work to understand.

### 4.2. Do Different Strains of the Fungus Differentially Moderate the Impacts of Elevated CO_2_ on the Growth and Seed Production of Perennial Ryegrass?

We did not find evidence of differences in the effects of the fungal strains on the impacts of elevated CO_2_ at the level of the whole-plant. There was no evidence of a CO_2_ × endophyte strain interaction for total plant biomass, seed number, or seed biomass (Figure 1). Multiple endophyte strains have not previously been studied in elevated CO_2_, but there are several studies comparing endophyte presence or absence in elevated CO_2_, in both the perennial ryegrass and tall fescue systems. In those studies, CO_2_× endophyte presence/absence interactions were similarly not observed for whole-plant responses [25,63,64,65]. While one cannot infer “no effect” from a failure to reject the null hypothesis, taken together, this study and the previous work suggest that perhaps endophyte strain and CO_2_ combine additively, at least in their effect on whole plant responses like biomass production. Nevertheless, this study, and the previous work on endophytes and elevated CO_2_, all suffer from a lack of statistical power due to the challenges of replication of the CO_2_ treatment. A lack of power means that only interactions with large effect sizes are likely to be detected in such experiments.

### 4.3. Are the Metabolomes of the Host Plant–Fungal Strain Combinations Different From Each Other and How Are They Altered by Elevated CO_2_?

From Figure 4 and Figure 5, and Table A1, it is clear that infection by the different strains of the endophyte resulted in many, sometimes quite large, differences in the host-fungus metabolome. In ambient CO_2_ (Figure 4), all of the comparisons except those involving NEA2 resulted in more than 100 metabolites for which the concentrations differed between strains. However, the differences between the strains were more “muted” at elevated CO_2_ (Figure 4). For example, AR37 vs. E+ in ambient CO_2_ resulted in 213 metabolite differences, whereas in elevated CO_2_ there were only 165 metabolite differences. Comparing within the same fungal strain in ambient CO_2_ vs. elevated CO_2_ we found only three metabolites with large differences.

While we were able to identify many differences in our untargeted metabolomics analysis, we were unfortunately not able to give names to many of these compounds. However, there were some intriguingly large differences in a handful of metabolites that probably warrant more targeted metabolomic analysis. In particular, there are three metabolites that, in ambient CO_2_, differ by >10-fold between AR1 and E+ as well as between AR37 and E+. One of these metabolites we were able to tentatively identify as Soyasaponin A2, a triterpenoid glycoside traditionally identified in soybeans. Soyasaponin A2 was clustered with six other related but unidentifiable compounds. At ambient CO_2_, all comparisons between fungal strains resulted in differences of 7 to 10-fold, with the exception of AR1 vs. AR37 (1.6-fold difference). These differences remained consistent at elevated CO_2_, except that there was no difference between AR1 and AR37.

Several compounds tentatively identified as alkaloids also showed important differences between fungal strains. Peramine (C_12_H_17_N_5_O) shows up often in the strain comparisons. Several of these entries involve comparisons with AR37, which is not surprising since AR37 does not produce peramine. However, peramine was also different in abundance between AR1 and E+, and between AR1 and NEA2, both in ambient CO_2_. Other tentatively identified alkaloids that showed differences included: 2-hydroxymethyl-4-methylquinazoline (C_10_H_10_N_2_O), paraherquamide E (≡ VM 54159, C_28_H_35_N_3_O_4_), the ergot alkaloid setoclavine (C_16_H_18_N_2_O), the tricyclic ergot alkaloid intermediate chanoclavine-I, and the transmembrane channel-like protein (TMC) 2B (C_28_H_34_N_4_O_8_). None of these latter alkaloids have previously been discussed in association with any of these endophyte strains. It is possible that this is a novel result or that we were unable to appropriately distinguish the LC-MS response curves. It is also possible that these metabolites are made by other, as yet uncharacterized, parts of the grass microbiome. Several other tenetatively identified secondary metabolites associated with herbivore defense also differed between strains. For example, the phenols 4-hydroxymellein (C_10_H_10_O_4_) and terphenyllin (≡ NSC 299114, C_20_H_18_O_5_) both differed. Other putative defensive metabolites for which differences were detected included benzyl benzoate (C_6_H_5_CH_2_O_2_CC_6_H_5_), MacFadienoside (C_15_H_22_O_11_), cucurbitacin (C_30_H_42_O), as well as the triterpene soyasaponin A mentioned earlier. Again, these putative defensive compounds are not known from the grass–*Epichloë* interaction and might possibly be products of other organisms from the grass’s microbiome. Lastly, it is also interesting to note that we detected differences in a compound tentatively identified as chlorogenic acid (C_16_H_18_O_9_), which is an intermediate in lignin biosynthesis.

In the one previous study of the metabolic impacts of elevated CO_2_ on the perennial ryegrass–*E. festucae* var. *lolii* interaction, Hunt et al. [25] found that endophyte-infected plants changed less under elevated CO_2_ than endophyte-free plants in terms of high molecular weight carbohydrates, soluble protein, and chlorophyll concentrations. While we did not include the endophyte-free plants in this experiment, a similar trend for muted responses to elevated CO_2_ was seen in our results. It is worth noting that the ambient CO_2_ treatment in the current study (400 ppm) was very similar to the elevated CO_2_ treatment in the Hunt et al. study (466 ppm). There have been a few studies of metabolic differences due to *E. coenophiala* endophyte in tall fescue (*S. arundinaceus* ≡ *F. arundinacea*) in response to elevated CO_2_. Newman et al. [63] found that tall fescue plants infected with the endophyte had smaller reductions in crude protein (%dry matter), smaller increases in soluble crude protein (%DM), and smaller reductions in acid detergent insoluble crude protein than did endophyte-free plants. Ryan et al. [64] found that low molecular weight carbohydrate concentrations increased more under elevated CO_2_ in endophyte-infected plants compared to endophyte-free plants and that peramine, ergovaline, and total lolines were all greater under elevated CO_2_. On the other hand, Brosi et al. [66] found that elevated CO_2_ caused declines in the concentrations of both ergovaline and loline, by *c*. 30%, suggesting that there is still work to be done to understand the responses of alkaloid production to rising CO_2_ concentrations. Ryan et al. [65] did not find any effects of the endophyte on phloem amino acid chemistry. Brosi et al. [66] found declines in cellulose, hemicellulose, and lignin due to CO_2_, but these differences were not related to the presence of the endophyte or the interaction between endophyte and CO_2_.

### 4.4. Are the Proteomes of the Host Plant–Fungal Strain Combinations Different From Each Other and How Are They Altered by Elevated CO_2_?

Between the PCA and the univariate analysis, we identified 192 proteins that indicate changes in abundance between ambient and elevated CO_2_. In both fungal and plant derived proteins, these changes in protein abundances occurred across a range of different functions, suggesting fairly widespread changes in protein synthesis.

In plants, SNARE protein function is known to restrict the growth of different pathogens; disruption of plant vesicle machinery may be indicative of microbial disturbance [88]. We observed an increase in SNARE protein production in AR1 and AR37 in elevated compared to ambient CO_2_ conditions, suggesting a plant response to endophyte growth under rising CO_2_ levels. In addition, we observed increased production of several vesicle-associated and transport proteins in the presence of AR1, which may correspond to the increased plant defense responses reported above. Plant defense responses may also be activated during fluctuations in transport. For example, engagement of plant ubiquitination-dependent proteasome machinery may result from pathogen attack [89] and therefore, we also investigated occurrences of proteolysis, protein ubiquitination, and protein folding, and we identified several proteins in AR1 and AR37 with increased production at 800 ppm CO_2_. For example, two ubiquitin-associated proteins showed increased abundance with AR1. These results suggest either increased protein degradation at elevated CO_2_ levels associated with stress response of the plant or increased degradation of plant proteins as a result of endophyte presence at elevated CO_2_ levels. Notably, AR37 also shows a reduction in proteasome production at elevated CO_2_ (suggesting a possible balance mechanism in effect).

Aside from differences in plant defense response proteins, we also observed unique production profiles of endophyte proteins. For example, AR1 and NEA2 showed the greatest number of proteins with changed abundances under elevated CO_2_ conditions with the majority of AR1 proteins demonstrating an increase in abundance, which may correspond with the increased bioprotective properties. Conversely, all different proteins identified with NEA2 were lower in abundance at 800 ppm CO_2_, including a pathogenesis-related protein (vesicle-associated membrane protein), which may support a decrease in fungal virulence at elevated CO_2_ and suggests a reduced stress response by the endophyte. For example, in the fungal pathogen *Candida albicans*, elevated CO_2_ levels enhance virulence during infection through regulation of signaling cascades, which suggests an opportunity for the fungus to flourish when the host’s immune system is suppressed [90].

### 4.5. Does an Integrated Analysis of the Proteome and Metabolome Data Yield Different Insights Than Those Gained From Considering the Proteome and Metabolome Separately?

By using the power of a multivariate analysis we are able to glean more information than is available from univariate analyses alone—because we use the information about relationships among the dependent variables not just between the independent and dependent variables. Similarly, by combining the *Epichloë* concentrations (abundances) with the metabolite and protein abundances, we are able to take advantage of information that is shared between the dependent variables. This analysis highlighted 34 metabolites and proteins that were not seemingly important in the separate PCAs or univariate analyses.

**Figure 9:**
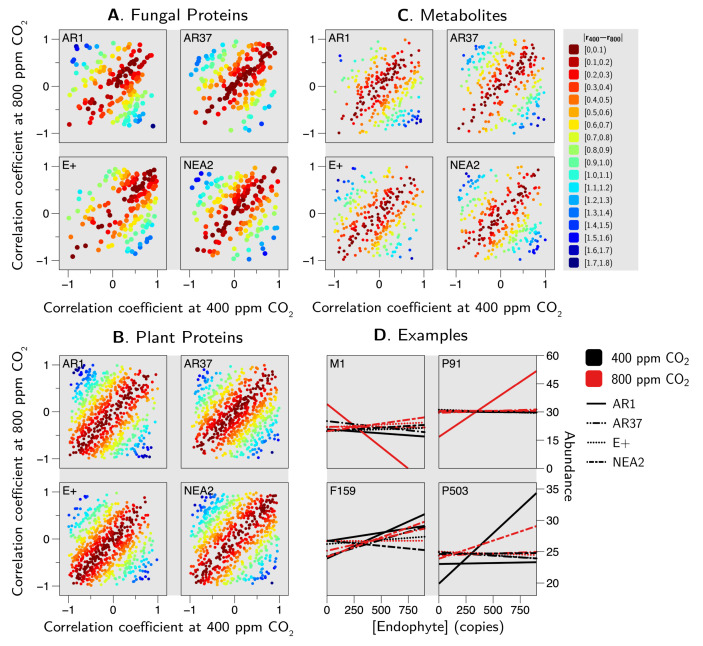
Correlations with *Epichloë* concentration. Show are the correlation coefficients (*r*) and the individual proteins (**A**,**B**) and metabolites (**C**) separated by epichoid strain (note that the points for the fungal proteins are larger for ease of viewing). Furthermore, (**D**) examples of the relationships between epichloid strain and four compounds (M1 ≡ “metabolite 1”, P91 ≡ “plant protein 91”, F159 ≡ “fungal protein 159”, P503 ≡ “plant protein 503” are shown; see Supplementary Tables S1 and S2) that are highly correlated (r>0.99) with at least one of the epichloid strain by CO_2_ combinations. Black lines represent 400 ppm CO_2_, red lines denote 800 ppm CO_2_; the different line types denote different ephichloid strains, but their identities are not important here. Here it suffices to note that the pattern of responses tends to be “strain specific” rather than general across all strains.

Taken together, the separate metabolome and proteome analyses, combined with the integrated OMICs analysis, allowed us to identify a large range of metabolites and proteins that seem to depend on the particular strain of the endophyte, the CO_2_ concentration, and often an interaction between the two. These results lay the groundwork for much follow-up research that will be necessary to elucidate the causal pathways and regulatory mechanisms that govern the host grass–fungal endophyte relationship (see “Conclusions and future directions” below). Doing so will not be easy. Although there are a large number of metabolites and proteins that vary among the treatments, there is no simple, consistent pattern of variation among the epichloid strains or across the CO_2_ treatments. To better illustrate this point, consider Figure 9. Shown are the correlation coefficients between the endophyte concentration and the various metabolites and proteins (for the subset of data for which we had metabolomic and proteomic measures). The coefficients at 400 ppm CO_2_ are plotted against the corresponding coefficient at 800 ppm. We can see that every possible relationship exists. Even for metabolites and proteins that show a very high correlation (r>0.99) for at least one of the strain–CO_2_ conditions (see Figure 9) the other strain–CO_2_ combinations show different patterns.

These differences in host-endophyte response between protein and metabolite production highlight the dynamic and complex regulatory processes underscoring the host and endophyte responses to infection and rising CO_2_ conditions. Similar differential profiles between proteomic and metabolomic data sets have been observed in diverse biological systems and may be explained by the different tissues used for the analysis or protein turnover rates [91]. For example, we performed metabolome profiling on blade tissue, whereas we performed the proteome profiling on pseudostem tissue, where fungal genes are more highly expressed [77] and where concentrations of the endophyte are greatest [85,92,93,94]. Such differences could be associated with the sample type, location, and possible diffusion of metabolites throughout the plant. To gain a better understanding of the relationship between protein and metabolite production, future studies should profile the metabolome of the pseudostem and use tandem mass spectrometry to identify compounds with greater accuracy. Another approach would be to use apoplast wash fluid, a technique used successfully by Green et al. [71] who noted that this approach greatly simplified the complex metabolomic response by limiting the metabolites to those likely produced by the endophyte.

Discrepancies between endophyte concentrations and protein abundance is a well-studied area and a lack of correlation is linked to several factors, including the intracellular stability of a protein (e.g., protein turnover rates), transcript stability, and post-translational regulation [95,96]. In addition, timing of sample collection and processing (e.g., early or late harvest), storage conditions (e.g., flash frozen vs. lyophilized), and tissue type (e.g., sheath tissue vs. pseudostem) can account for differences in molecular regulation. Moreover, changing patterns in protein translation and transcription can provide insight into differences in gene regulation.

### 4.6. Is There Any Evidence of Host Plant–Fungal Strain Genetic Incompatibility?

We found no evidence of genetic incompatibility. The between family variance was considerably smaller than the within family variance in the three plant growth metrics as well as the endophyte concentration measure. It is unclear how the genetic variation in our plants would compare to the genetic variation in other, nonagronomic grass–*Epichloë* combinations. Recall that we purposefully created additional genetic variation in the host plants by outcrossing the mothers with a variety of other forage and turf cultivars. Nevertheless, it remains an open question whether our procedure could have created sufficient variation to detect genetic incompatibility in this plant–fungal interaction. The relationship between cool season grasses and *Epichloë* endophytes is an ancient one, arising some 30–40 million years ago. It seems to us unlikely that genetic incompatibility between host grass and *Epichloë* endophyte still persists, but our test is by no means definitive.

## 5. Conclusions and Future Directions

In brief, we asked and tentatively answered the following questions:

1. Do different strains of E. festucae var. lolii produce similar fungal concentrations in a genetically diverse host plant background?
  No, in our experiment AR37 produced greater concentrations of the endophyte than did any of the other strains (Figure 1).
2. Do different strains of the fungus differentially moderate the impacts of elevated CO_2_ on the growth and seed production of perennial ryegrass?
  No, we did not find evidence that endophyte strains interact with CO_2_ to influence plant growth or seed production (Figure 1).
3. Are the metabolomes of the host plant–fungal strain combinations different from each other and how are they altered by elevated CO_2_?
  Yes, metabolomes differed between endophyte strains and these differences were generally more muted in elevated CO_2_ compare to ambient CO_2_ (Table A1, Figure 4 and Figure 5).
4. Are the proteomes of the host plant–fungal strain combinations different from each other and how are they altered by elevated CO_2_?
  Yes, proteomes differed between endophyte strains and there was evidence of substantial interaction between endophyte strains and CO_2_ levels (Table A2, Table A3 and Table A4, Figure 6 and Figure 7).
5. Does an integrated analysis of the proteome and metabolome data yield different insights than those gained from considering the proteome and metabolome separately?
  Yes, the integrated analysis highlighted roles for 34 metabolites and proteins that were not identified as important in the previous analyses (Table A5, Figure 8).
6. Is there any evidence of host plant–fungal strain genetic incompatibility?
  No, we found no evidence of genetic incompatibility for the degree of genetic diversity we were able to create in this experiment (Figure 2).

The OMICs techniques we employed in this paper are, in some sense, like opening the “black box” that is not visible in studies of the *Epichloë*–grass relationship studied only at the level of gross plant growth and reproduction metrics. In many ways, the work generates more questions than answers. It might be productive to build upon this work in the following ways. To get a better sense of the impacts of climatic change on this mutualism, a future experiment ought to incorporate warming and the interaction between warming and elevated CO_2_. The metabolomic impacts of endophyte strain and climatic change ought to be assessed using tandem mass spectrometry (LC-MS/MS) to gain sensitivity and structural information to better identify metabolites. A more targeted metabolomics approach, using internal standards, would also be warranted to explore in more depth some of the changes we observed in the present experiment. A useful extension of our proteomics analysis would be to examine in more depth some of the protein abundance changes we observed, perhaps coupled with a targeted transcriptomic assay [97]. Finally, although we recognize that this would be a major undertaking, work should be done to identify the entire plant microbiome, how it changes with different epichloid strains, and its functional role in altering the plant growth, metabolomic, and proteomic responses to climatic change. In terms of the genetic incompatibility question, a similar experiment using families of half-sibs needs to be completed on nonagricultural populations of grass-endophyte combinations.

## Figures and Tables

**Table 1:** Sample size of plants nested in family, nested in endophyte strain. There were 288 plants in total, 144 in each of the two levels of CO_2_. Within a family there are 24 plants, 12 in each level of CO_2_. The families are replicated in each level of CO_2_. For the proteome analysis we used 5 of the 12 plants in each level of CO_2_ from a single family per endophyte strain; these are denoted with the red font.

Strain	400 ppm	800 ppm	Measurements
**AR1**			
Family *a*	$p_{1},\ldots ,p_{5}$	$p_{13},\ldots ,p_{17}$	Biomass, qPCR, proteomics,
	$p_{6},\ldots ,p_{12}$	$p_{18},\ldots ,p_{24}$	metabolomics, integrated OMICS
Family *b*	$p_{1},\ldots ,p_{12}$	$p_{13},\ldots ,p_{24}$	Biomass, qPCR, metabolomics
Family *c*	$p_{1},\ldots ,p_{12}$	$p_{13},\ldots ,p_{24}$	Biomass, qPCR, metabolomics
Family *d*	$p_{1},\ldots ,p_{12}$	$p_{13},\ldots ,p_{24}$	Biomass, qPCR, metabolomics
**AR37**			
Family *e*	$p_{1},\ldots ,p_{5}$	$p_{13},\ldots ,p_{17}$	Biomass, qPCR, proteomics,
	$p_{6},\ldots ,p_{12}$	$p_{18},\ldots ,p_{24}$	metabolomics, integrated OMICS
Family *f*	$p_{1},\ldots ,p_{12}$	$p_{13},\ldots ,p_{24}$	Biomass, qPCR, metabolomics
Family *g*	$p_{1},\ldots ,p_{12}$	$p_{13},\ldots ,p_{24}$	Biomass, qPCR, metabolomics
**E+**			
Family *h*	$p_{1},\ldots ,p_{5}$	$p_{13},\ldots ,p_{17}$	Biomass, qPCR, proteomics,
	$p_{6},\ldots ,p_{12}$	$p_{18},\ldots ,p_{24}$	metabolomics, integrated OMICS
Family *i*	$p_{1},\ldots ,p_{12}$	$p_{13},\ldots ,p_{24}$	Biomass, qPCR, metabolomics
Family *j*	$p_{1},\ldots ,p_{12}$	$p_{13},\ldots ,p_{24}$	Biomass, qPCR, metabolomics
**NEA2**			
Family *k*	$p_{1},\ldots ,p_{5}$	$p_{13},\ldots ,p_{17}$	Biomass, qPCR, proteomics,
	$p_{6},\ldots ,p_{12}$	$p_{18},\ldots ,p_{24}$	metabolomics, integrated OMICS
Family *l*	$p_{1},\ldots ,p_{12}$	$p_{13},\ldots ,p_{24}$	Biomass, qPCR, metabolomics
**Totals**	144 plants	144 plants	

## Supplementary Materials

The following are available online at https://www.mdpi.com/2309-608X/6/4/360/s1, Figure S1: Biological replicate reproducibility, Table S1: Metabolite univariate differences, Table S2: Summary of metabolite differences.

## Appendix A. Detailed Metabolomics Results

**Table A1:** Metabolites defining the first two principal components. Shown are the metabolites that “load heavily” (in this case |x|≥0.7) on each axis. In the case of PC1, these metabolites load positively. In the case of PC2, the metabolites load negatively. $\bar{\mathrm{Mass}}$ denotes the average mass, and $\bar{\mathrm{RT}}$ denotes the average retention time. Metabolites that also loaded heavily in the integrated OMICs PCA are denoted with a ✓, see Table A5. See also Supplemental Tables S1 and S2.

1st Principal Component			
$\bar{\mathrm{Mass}}$	$\bar{\mathrm{RT}}$	Compound Name	Loading
404.20	1.26	unidentified #121	0.92
1049.53	5.09	unidentified #278	0.89
893.44	4.45	unidentified #269	0.89
236.13	4.71	unidentified #34	0.88
477.26	4.56	✓ VM 54159	0.87
480.89	5.15	unidentified #153	0.87
481.55	5.15	unidentified #155	0.86
1078.52	4.98	unidentified #280	0.86
1148.60	5.15	unidentified #289	0.84
121.05	5.15	✓ unidentified #3	0.83
514.28	5.16	Cucurbitacin I	0.82
992.47	5.15	unidentified #275	0.82
891.46	5.12	unidentified #268	0.82
1106.56	5.16	✓ Soyasaponin A2	0.81
368.85	5.17	✓ unidentified #91	0.80
1122.55	4.45	✓ unidentified #283	0.80
374.18	4.45	unidentified #96	0.80
860.42	5.17	unidentified #263	0.79
1120.57	5.16	unidentified #282	0.79
373.85	5.17	unidentified #95	0.79
**2st Principal Component**			
$\bar{\mathrm{Mass}}$	$\bar{\mathrm{RT}}$	**Compound Name**	**Loading**
212.08	5.14	Benzyl benzoate	0.79
374.85	4.44	unidentified #98	0.78
256.16	4.75	Chanoclavine-I	0.77
1498.71	5.15	unidentified #301	0.77
645.33	5.15	unidentified #214	0.74
480.56	5.15	unidentified #152	0.74
514.31	1.22	unidentified #164	0.74
1458.69	5.15	unidentified #300	0.73
878.37	5.16	unidentified #266	0.70
**2nd Principal Component**			
$\bar{\mathrm{Mass}}$	$\bar{\mathrm{RT}}$	**Compound Name**	
364.14	7.51	unidentified #86	−0.70
348.11	5.75	unidentified #75	−0.71
332.12	5.32	unidentified #69	−0.72
316.08	5.31	unidentified #61	−0.73
362.09	4.70	unidentified #84	−0.73
396.13	6.05	unidentified #115	−0.73
378.12	6.04	Macfadienoside	−0.75
346.13	7.50	3-Methoxyxanthocillin X dimethyl ether	−0.75
558.18	5.02	unidentified #182	−0.78
315.21	5.29	unidentified #310	−0.81
396.13	6.05	unidentified #115	−0.81
142.06	6.02	unidentified #6	−0.82
558.18	5.08	unidentified #181	−0.85
334.13	8.61	unidentified #71	−0.86
302.07	5.30	unidentified #55	−0.86

## Appendix B. Detailed Proteomics Results

**Table A2:** Proteome Principal Components Analysis. Shown are the proteins that “load heavily” onto the first three principal component axes. “Heavy” loadings are defined here as |x|≥0.70. Fungal derived proteins are highlighted in pale brown; the plant derived proteins are highlighted in pale green. Proteins that were individually different between 400 ppm and 800 ppm CO_2_ for at least one strain of the endophyte are denoted with a †, see Table A3 and Table A4. Proteins that load heavily in the integrated OMICs PCA are denoted with ✓, see Table A5.

	PCA Loadings		
**Protein**	**PC1**	**PC2**	**PC3**
✓ probable methionine synthase	0.85		
6-phosphogluconate dehydrogenase, decarboxylating	0.84		
FAD dependent oxidoreductase	0.80		
✓γ-actin	0.78		
hypothetical protein	0.78		
heat shock protein	0.75		
probable nucleoside-diphosphate kinase	0.75		
✓ related to sporulation-specific gene SPS2	0.75		
† 7α-cephem-methoxylase P8 chain related protein	0.75		
Iso_dh domain-containing protein	0.74		
60S ribosomal protein L13	0.73		
† Saccharopine dehydrogenase	0.72		
unnamed protein product	−0.71		
peptidyl-prolyl cis-trans isomerase CYP20-1 isoform X2	−0.71		
GTP-binding protein	−0.72		
dipeptidase	−0.72		
proteasome subunit α type-7-B	−0.72		
citrate synthase	−0.73		
protein TPR2	−0.74		
† transmembrane 9 superfamily member	−0.75		
glucose-6-phosphate isomerase	−0.75		
predicted protein	−0.76		
zinc protease	−0.77		
guanosine nucleotide diphosphate dissociation inhibitor	−0.80		
✓ unnamed protein product / MPN domain-containing protein		0.86	
✓† plant SNARE 13		0.86	
✓ pyruvate kinase, cytosolic isozyme		0.86	
✓† endoglucanase		0.86	
chromatin assembly factor 1 subunit A isoform X1		0.84	
unnamed protein product / Importin N-terminal domain-containing protein		0.83	
vesicle-associated protein		0.81	
† N-acetyl-D-glucosamine kinase		0.80	
✓ GTP-binding protein SAR1A		0.80	
uncharacterized protein		0.80	
predicted protein		0.79	
ATP-dependent Clp protease proteolytic subunit		0.78	
✓β-adaptin-like protein		0.75	
elongation factor 1-β		0.75	
† cytosolic acetyl-CoA carboxylase 2		0.75	
unnamed protein product / SRP54 domain-containing protein		0.75	
uncharacterized protein		0.74	
✓† histidine–tRNA ligase		0.73	
peroxisomal acyl-coenzyme A oxidase		0.73	
putative 6-phosphogluconolactonase 4		0.73	
† chloroplast protoporphyrinogen IX oxidase		0.72	
40S ribosomal protein S20		0.72	
† 60S ribosomal protein L10a		0.71	
hypothetical protein		0.71	
✓† ubiquitin carboxyl-terminal hydrolase		0.70	
✓ Ras family protein			0.79
✓ probable glutathione peroxidase			0.77
✓ 14-3-3E			0.77
putative glycerophosphoryl diester phosphodiesterase			0.75
fructose-bisphosphate aldolase			0.75
ran-binding protein			0.74
✓ vacuolar proton-inorganic pyrophosphatase			0.74
hypothetical protein			0.73
protein CROWDED NUCLEI			0.71
probable ribosomal protein			0.71
polyketide synthase			−0.73
† 60S ribosomal protein L32-1			−0.73
oxygen-dependent coproporphyrinogen-III oxidase			−0.81
uncharacterized protein			−0.81

**Table A3:** Differences in fungal proteins identified by quantitative proteomics profiling influenced by rising CO_2_ levels. Proteins that load heavily in the proteome PCA are denoted with a †, see Table A2. Proteins that load heavily in the integrated OMICs PCA are denoted with a ✓.

		LFQ (log_2_; 800 vs. 400 ppm)			
Gene Identifier	Protein Name	AR1	AR37	E+	NEA2
**GOBP: Metabolism**					
E2368|EfP3.074780.mRNA-1	dihydrolipoamide acetyltransferase	1.91			
E2368|EfP3.082070.mRNA-1	probable β-glucosidase 1 precursor	1.73			
E2368|EfP3.025570.mRNA-1	† saccharopine dehydrogenase				−2.16
E2368|EfP3.029340.mRNA-1	LysM domain-containing protein				−1.61
E2368|EfP3.034550.mRNA-1	probable H+-transporting ATPase	1.94			
E2368|EfP3.040190.mRNA-1	glycoside hydrolase family 10 protein	2.57			−2.24
E2368|EfP3.046900.mRNA-1	glyceraldehyde-3-phosphate dehydrogenase	−1.60			
E2368|EfP3.005420.mRNA-1	glycoside hydrolase family 3 protein	−2.46			
E2368|EfP3.059150.mRNA-1	inorganic pyrophosphatase	−2.25			
E2368|EfP3.015680.mRNA-1	α-mannosidase	−1.68			
E2368|EfP3.019300.mRNA-1	adenosylhomocysteinase		−2.39		
E2368|EfP3.019390.mRNA-1	probable trehalase precursor	−2.86			
E2368|EfP3.027680.mRNA-1	Acyl-CoA-binding protein	−2.97	−2.08		
E2368|EfP3.043990.mRNA-2	probable pyruvate decarboxylase	−1.66			
**GOBP: Biosynthetic & Catabolic processes**					
E2368|EfP3.011820.mRNA-1	cobalamin-independent methionine synthase				−2.15
E2368|EfP3.002240.mRNA-1	argininosuccinate lyase				1.53
E2368|EfP3.032860.mRNA-1	3-isopropylmalate dehydrogenase	1.91			−1.97
E2368|EfP3.064110.mRNA-2	probable phosphogluconate dehydrogenase				−1.90
**GOBP: Translation & Transcription**					
E2368|EfP3.056000.mRNA-1	40S ribosomal protein S15				−2.15
E2368|EfP3.066450.mRNA-1	probable ribosomal protein L12	6.22	3.71		
E2368|EfP3.011650.mRNA-1	40S ribosomal protein S0				−2.04
E2368|EfP3.046770.mRNA-1	40S ribosomal protein S7				−2.60
E2368|EfP3.020500.mRNA-1	ribonuclease HI large subunit	−2.89	−4.47		
E2368|EfP3.026100.mRNA-1	Histone H2B	−2.20			
**GOBP: Uncharacterized**					
E2368|EfP3.004630.mRNA-1	† 7α-cephem-methoxylase P8 chain related protein	3.81			-4.95
E2368|EfP3.014290.mRNA-1	endosomal peripheral membrane protein	1.83			
E2368|EfP3.053290.mRNA-1	uncharacterized protein	−2.29			
E2368|EfP3.057600.mRNA-1	hypothetical protein	−2.44			
E2368|EfP3.080080.mRNA-1	WD repeat protein		−3.04		
**GOBP: Protein folding**					
E2368|EfP3.079510.mRNA-1	40 kDa peptidyl-prolyl cis-trans isomerase				−5.12
E2368|EfP3.059210.mRNA-1	calreticulin	−1.86			
**GOBP: Pathogenesis**					
E2368|EfP3.079280.mRNA-1	Vesicle-associated membrane protein				−1.80
**GOBP:: Aerobic respiration**					
E2368|EfP3.007970.mRNA-1	cytochrome b-c1 complex subunit 2	2.14			−1.79
**GOBP: Genome maintenance**					
E2368|EfP3.031010.mRNA-1	ATP citrate lyase	2.05			−1.90
**GOBP: DNA binding**					
E2368|EfP3.059770.mRNA-1	cold-shock DNA-binding domain-containing protein		−3.00		

**Table A4:** Differences in plant proteins identified by quantitative proteomics profiling influenced by rising CO_2_ levels. Proteins that load heavily in the proteome PCA are denoted with a †, see Table A2. Proteins that load heavily in the integrated OMICs PCA are denoted with a ✓, see Table A5.

		LFQ (log_2_; 800 vs. 400 ppm)			
Gene Identifier	Protein Name	AR1	AR37	E+	NEA2
**GOBP: Translation & RNA processing**					
ref0006279-exonerate_est2genome-gene-0.0-mRNA-1	sm-like protein	2.69			
ref0046235-exonerate_est2genome-gene-0.1-mRNA-1	30S ribosomal protein 3				−1.95
ref0003115-exonerate_est2genome-gene-0.3-mRNA-1	✓† histidine–tRNA ligase	1.38			
ref0012853-exonerate_est2genome-gene-1.5-mRNA-1	RGG repeats nuclear RNA binding protein A-like	2.12			
ref0029525-exonerate_est2genome-gene-0.5-mRNA-1	50S ribosomal protein L31	3.30			
ref0002751-exonerate_est2genome-gene-0.0-mRNA-1	eukaryotic translation initiation factor 4G	1.90			
ref0037446-exonerate_est2genome-gene-0.1-mRNA-1	† 60S ribosomal protein L10a	4.72			
ref0039514-exonerate_est2genome-gene-0.1-mRNA-1	valine–tRNA ligase	1.26			−1.76
ref0005003-exonerate_est2genome-gene-0.0-mRNA-3	serine/arginine-rich-splicing factor SR34 isoform	2.33			
ref0004691-exonerate_est2genome-gene-0.6-mRNA-1	DEAD-box ATP-dependent RNA helicase 3	1.58			
ref0026558-exonerate_est2genome-gene-0.0-mRNA-2	eukaryotic translation initiation factor 6-2				−2.04
ref0005830-exonerate_est2genome-gene-0.0-mRNA-1	small nuclear ribonucleoprotein SmD1a	1.63			−1.98
ref0031460-exonerate_est2genome-gene-0.1-mRNA-1	splicing factor 3B subunit 1	1.71			−1.73
ref0002750-exonerate_est2genome-gene-0.0-mRNA-1	50S ribosomal protein L29			−2.82	−2.59
ref0027372-exonerate_est2genome-gene-0.0-mRNA-1	† 60S ribosomal protein L32-1		−2.37		
ref0024485-exonerate_est2genome-gene-0.5-mRNA-1	DEAD-box ATP-dependent RNA helicase 20	−1.71			
ref0020192-exonerate_est2genome-gene-0.1-mRNA-1	nardilysin-like	−1.85			
ref0047393-exonerate_est2genome-gene-0.0-mRNA-1	translation initiation factor IF3-4	1.92			
ref0005329-exonerate_est2genome-gene-0.0-mRNA-2	✓ glycine–tRNA ligase	1.54			
**GOBP: Biosynthetic & catabolic processes**					
ref0029850-exonerate_est2genome-gene-0.0-mRNA-1	† chloroplast protoporphyrinogen IX oxidase 1	1.56			
ref0045266-exonerate_est2genome-gene-0.0-mRNA-1	Cytochrome P450	3.02	2.73		−2.45
ref0014716-exonerate_est2genome-gene-0.1-mRNA-1	glutamyl-tRNA(Gln) amidotransferase subunit C	2.04			
ref0040294-exonerate_est2genome-gene-0.0-mRNA-2	lipoamide acyltransferase	2.40			−2.67
ref0010297-exonerate_est2genome-gene-0.2-mRNA-1	glutamate–glyoxylate aminotransferase 2 isoform	1.60			
ref0029399-exonerate_est2genome-gene-0.0-mRNA-1	trehalose-6-phosphate synthase	2.32			
ref0008372-exonerate_est2genome-gene-0.1-mRNA-1	phospholipase A1-II 7	2.15			
ref0020040-exonerate_est2genome-gene-0.1-mRNA-1	δ-aminolevulinic acid dehydratase	2.62			−2.22
ref0041371-exonerate_est2genome-gene-0.0-mRNA-1	12-oxophytodienoate reductase 11	2.25			
ref0026877-exonerate_est2genome-gene-0.0-mRNA-1	linoleate 9S-lipoxygenase 3			3.43	
ref0042665-exonerate_est2genome-gene-0.2-mRNA-1	endoglucanase 24	3.13	1.98		
ref0023177-exonerate_est2genome-gene-1.6-mRNA-1	pyruvate dehydrogenase E1 component subunit α-3	1.71			
ref0046445-exonerate_est2genome-gene-0.3-mRNA-1	chloroplast stem-loop binding protein of 41 kDa b	1.69			−1.61
ref0025738-exonerate_est2genome-gene-0.0-mRNA-1	putative monogalactosyldiacylglycerol synthase 1	2.03	2.12		
ref0013554-exonerate_est2genome-gene-0.5-mRNA-1	aldehyde oxidase 2	−1.42			
ref0032452-exonerate_est2genome-gene-0.2-mRNA-1	protein ECERIFERUM 26-like		−2.13		
ref0000037-exonerate_est2genome-gene-0.2-mRNA-1	alcohol dehydrogenase 4	−1.34			
ref0043257-exonerate_est2genome-gene-0.0-mRNA-1	cinnamyl alcohol dehydrogenase				2.02
**GOBP: Transport**					
ref0016004-exonerate_est2genome-gene-0.3-mRNA-1	✓† Plant SNARE 13	2.87	1.62		
ref0007462-exonerate_est2genome-gene-0.1-mRNA-2	cation-chloride cotransporter 1-like isoform X2				−1.84
ref0029020-exonerate_est2genome-gene-0.0-mRNA-1	γ-soluble NSF attachment protein	1.73			
ref0006742-exonerate_est2genome-gene-0.0-mRNA-2	plasma membrane ATPase 1				−1.73
ref0036493-exonerate_est2genome-gene-0.3-mRNA-1	exportin-2	2.70			
ref0009047-exonerate_est2genome-gene-0.0-mRNA-2	✓† vesicle-associated protein 1-3-like	2.47	2.09		
ref0017192-exonerate_est2genome-gene-0.0-mRNA-1	vacuolar targeting receptor bp-80	1.64			
ref0004589-exonerate_est2genome-gene-0.0-mRNA-1	ABC transporter F family member 1-like	1.87			
ref0032029-exonerate_est2genome-gene-0.1-mRNA-3	† transmembrane 9 superfamily member 12-like	2.36			
ref0006339-exonerate_est2genome-gene-0.4-mRNA-1	importin subunit β-1-like	1.43			
**GOBP: Defense response**					
ref0030923-exonerate_est2genome-gene-0.2-mRNA-1	aspartyl protease family protein 1	2.16			
ref0035348-exonerate_est2genome-gene-0.1-mRNA-1	primary amine oxidase 1	1.77			
ref0043342-exonerate_est2genome-gene-1.4-mRNA-1	AIG2-like protein D	1.63			
ref0014914-exonerate_est2genome-gene-0.0-mRNA-1	tryptophan synthase β chain 2		2.35		
ref0036720-exonerate_est2genome-gene-0.2-mRNA-2	endo-1,3(4)-β-glucanase 2	1.61			
ref0029599-exonerate_est2genome-gene-0.3-mRNA-1	peroxidase 1-like	1.85			
ref0009434-exonerate_est2genome-gene-0.0-mRNA-1	peroxidase 43-like	1.57			
ref0032529-exonerate_est2genome-gene-0.0-mRNA-1	peroxidase 47-like	1.97			
ref0038358-exonerate_est2genome-gene-0.3-mRNA-1	pathogen-related protein 10-3	2.63			
ref0000436-exonerate_est2genome-gene-0.1-mRNA-1	protein DJ-1 homolog A	1.92			
ref0042726-exonerate_est2genome-gene-0.0-mRNA-1	ferritin-1		−3.07		
ref0046713-exonerate_est2genome-gene-0.1-mRNA-1	Glucan endo-1,3-β-glucosidase 4	−1.76			
ref0041974-exonerate_est2genome-gene-0.0-mRNA-1	nonspecific lipid transfer protein-like 1		−3.04		
ref0022536-exonerate_est2genome-gene-0.1-mRNA-1	metacaspase 3	−1.73			
**GOBP: Uncharacterized**					
ref0011040-exonerate_est2genome-gene-0.0-mRNA-1	uncharacterized protein		2.05		
ref0007943-exonerate_est2genome-gene-0.4-mRNA-3	hypothetical protein				−2.04
ref0026121-exonerate_est2genome-gene-0.3-mRNA-1	hypothetical protein	1.99			−3.23
ref0036333-exonerate_est2genome-gene-0.0-mRNA-1	unnamed protein product	2.65			−2.53
ref0025567-exonerate_est2genome-gene-0.1-mRNA-1	✓ hypothetical protein	1.54			
ref0034929-exonerate_est2genome-gene-0.0-mRNA-1	uncharacterized protein	2.05			
ref0040401-processedgene-0.3-mRNA-1	hypothetical protein	1.96			
ref0038111-exonerate_est2genome-gene-0.0-mRNA-1	unnamed protein product		−1.77		
ref0018438-exonerate_est2genome-gene-0.0-mRNA-2	large proline-rich protein bag6-B isoform	−2.03			
ref0044732-processed-gene-0.3-mRNA-1	predicted protein		−2.01		
**GOBP: Metabolism**					
ref0036350-exonerate_est2genome-gene-0.2-mRNA-1	aldo-keto reductase family 4 member C10	1.81			−1.98
ref0046846-exonerate_est2genome-gene-0.2-mRNA-1	putative aldo-keto reductase 2	2.74			
ref0036721-exonerate_est2genome-gene-0.0-mRNA-2	sphingosine-1-phosphate lyase	2.98			
ref0037951-exonerate_est2genome-gene-0.0-mRNA-1	Glu1 protein				−2.67
ref0021220-exonerate_est2genome-gene-0.2-mRNA-1	methylcrotonoyl-CoA carboxylase subunit α	1.59			
ref0015207-exonerate_est2genome-gene-0.0-mRNA-1	α-L-arabinofuranosidase 1-like	1.56			−2.84
ref0042878-exonerate_est2genome-gene-0.0-mRNA-2	† acyl-coenzyme A oxidase 2	2.34			
ref0040981-exonerate_est2genome-gene-0.1-mRNA-1	UDP-N-acetylglucosamine diphosphorylase 1		−2.71		
**GOBP: Cell cycle & development**					
ref0006993-exonerate_est2genome-gene-0.0-mRNA-1	dynamin-related protein 1E	1.94			
ref0040384-exonerate_est2genome-gene-0.2-mRNA-1	myosin-17-like		1.92		
ref0013885-snap-gene-0.15-mRNA-1	early nodulin-like protein 1	2.01	2.83		
ref0032994-exonerate_est2genome-gene-0.0-mRNA-2	† chromatin assembly factor 1 subunit A isoform	2.50			
ref0042157-exonerate_est2genome-gene-0.4-mRNA-1	probable cellulose synthase A catalytic subunit 8	3.07			
ref0033306-exonerate_est2genome-gene-0.0-mRNA-1	NADH dehydrogenase [ubiquinone] 1 α subcomplex subunit	2.03			
ref0034289-exonerate_est2genome-gene-0.1-mRNA-1	† cytosolic acetyl-CoA carboxylase 2	2.04			
ref0003264-exonerate_est2genome-gene-0.0-mRNA-2	protein RCC2	−1.75			
ref0025755-exonerate_est2genome-gene-0.0-mRNA-1	Protein phosphatase 1 regulatory subunit		−2.03		
**GOBP: Proteolysis, protein ubiquitination & protein folding**					
ref0044837-exonerate_est2genome-gene-0.2-mRNA-1	ATP-dependent zinc metalloprotease	1.40	1.68		
ref0019120-exonerate_est2genome-gene-0.0-mRNA-3	✓ ubiquitin carboxyl-terminal hydrolase 13	2.33			−1.72
ref0026335-exonerate_est2genome-gene-0.1-mRNA-1	ubiquitin conjugation factor	2.57			−2.66
ref0025653-exonerate_est2genome-gene-1.2-mRNA-1	proteasome subunit α type-5				−1.54
ref0043339-exonerate_est2genome-gene-1.2-mRNA-1	dnaJ protein P58IPK homolog B isoform X1		2.05		
ref0035568-exonerate_est2genome-gene-0.2-mRNA-1	proteasome subunit β type-3		−1.56		
**GOBP: Signal transduction**					
ref0037043-exonerate_est2genome-gene-0.0-mRNA-1	† N-acetyl-D-glucosamine kinase	3.38	2.48		−1.68
ref0012852-exonerate_est2genome-gene-0.1-mRNA-1	nicalin	1.20			
ref0030401-exonerate_est2genome-gene-0.0-mRNA-1	signal recognition particle 54 kDa protein	1.86			
ref0013500-exonerate_est2genome-gene-0.0-mRNA-1	signal recognition particle subunit SRP72	2.53			

## Appendix C. Detailed Integrated OMICs Results

**Table A5:** Integrated OMICs Principal Components Analysis. Shown are the metabolites and proteins that “load heavily” onto the first four principal component axes. “Heavy” loadings are defined here as |x|≥0.75 for the proteins and |x|≥0.65 for the metabolites. Fungal derived proteins are highlighted in pale brown, plant derived proteins are highlighted in pale green, metabolites are highlighted in pale yellow, and the concentration of the *Epichloë* endophyte is highlighted in pale blue. Where metabolites or proteins are important in other analyses they are cross referenced to the relevant table.

	PCA Loadings			
**Identity**	**PC1**	**PC2**	**PC3**	**PC4**
related to sporulation-specific gene SPS2 (Table A2)	−0.85			
Superoxide dismutase [Cu-Zn]	−0.84			
probable methionine synthase (Table A2)	−0.79			
unidentified protein	−0.79			
γ-actin (Table A2)	−0.78			
related to gluconate 5-dehydrogenase	−0.77			
unidentified protein	−0.76			
uncharacterized proetin	−0.75			
$\bar{\mathrm{Mass}}=189.04$; $\bar{\mathrm{RT}}=3.81$; unidentified #19	−0.70			
*Epichloë fesctucae* var. *lolii* concentration (Figure 1)	−0.62			
$\bar{\mathrm{Mass}}=1106.56$; $\bar{\mathrm{RT}}=3.13$; Soyasaponin A2 (Table A1)	0.68			
$\bar{\mathrm{Mass}}=1122.55$; $\bar{\mathrm{RT}}=4.45$; unidentified #283 (Table A1)	0.69			
$\bar{\mathrm{Mass}}=368.85$; $\bar{\mathrm{RT}}=5.17$; unidentified #91 (Table A1)	0.75			
phospho-2-dehydro-3-deoxyheptonate aldolase 2, chloroplastic	0.75			
40S ribosomal protein S5-1	0.76			
26S proteasome non-ATPase regulatory subunit 1 homolog	0.76			
uncharacterized proetin	0.77			
uncharacterized proetin	0.79			
26S protease regulatory subunit 8 homolog A-like	0.80			
GDP-mannose 3,5-epimerase 2	0.86			
$\bar{\mathrm{Mass}}=328.20$; $\bar{\mathrm{RT}}=1.21$; unidentified #65		−0.72		
$\bar{\mathrm{Mass}}=477.26$; $\bar{\mathrm{RT}}=4.56$; VM54159 (Table A1)		−0.69		
$\bar{\mathrm{Mass}}=121.05$; $\bar{\mathrm{RT}}=5.15$; unidentified #3 (Table A1)		−0.65		
DNAJ-like protein		0.76		
RanBD1 domain-containing protein		0.77		
probable glutathione peroxidase 4 (Table A2)		0.77		
vacuolar proton-inorganic pyrophosphatase (Table A2)		0.78		
cytosolic copper zinc superoxide dismutase		0.79		
putative ADP-ribosylation factor		0.81		
14-3-3E (Table A2)		0.81		
Ras family protein (Table A2)		0.87		
U-box domain-containing protein			0.75	
uncharacterized protein			0.77	
glycine–tRNA ligase, chloroplastic/mitochondrial 2-like (Table A4)			0.78	
aldo_ket_red domain-containing protein			0.79	
histidine–tRNA ligase, cytoplasmic (Table A2 and Table A4)			0.79	
protoporphyrinogen oxidase			0.79	
uncharacterized protein			0.80	
uncharacterized protein (Table A2 and Table A4)			0.80	
acetyltransferase component of pyruvate dehydrogenase complex			0.80	
importin N-terminal domain-containing protein			0.81	
β-adaptin-like protein (Table A2)			0.82	
MI domain-containing protein			0.82	
ubiquitin carboxyl-terminal hydrolase 13 (Table A2 and Table A4)			0.82	
pyruvate kinase, cytosolic isozyme (Table A2)			0.83	
MPN domain-containing protein (Table A2)			0.84	
GTP-binding protein SAR1A (Table A2)			0.86	
vesicle-associated protein 1-3-like (Table A4)			0.86	
predicted protein			0.87	
uncharacterized protein			0.89	
UBA domain-containing protein			0.91	
endoglucanase (Table A2)			0.92	
plant SNARE 13 (Table A2 and Table A4)			0.93	
hypothetical protein IFM46972_10396				−0.81
β expansin B2				−0.77
$\bar{\mathrm{Mass}}=820.45$; $\bar{\mathrm{RT}}=6.72$; unidentified #249				−0.74
$\bar{\mathrm{Mass}}=834.47$; $\bar{\mathrm{RT}}=7.04$; unidentified #254				−0.70
uncharacterized protein				0.77
