# Supplementary figures and images for: Examining the Impacts of CO2 Concentration and Genetic Compatibility on Perennial Ryegrass—Epichloë festucae var lolii Interactions
Source: J Fungi (Basel). 2020 Dec 11;6(4):360. doi: 10.3390/jof6040360 (PMC7770580; doi:10.3390/jof6040360)

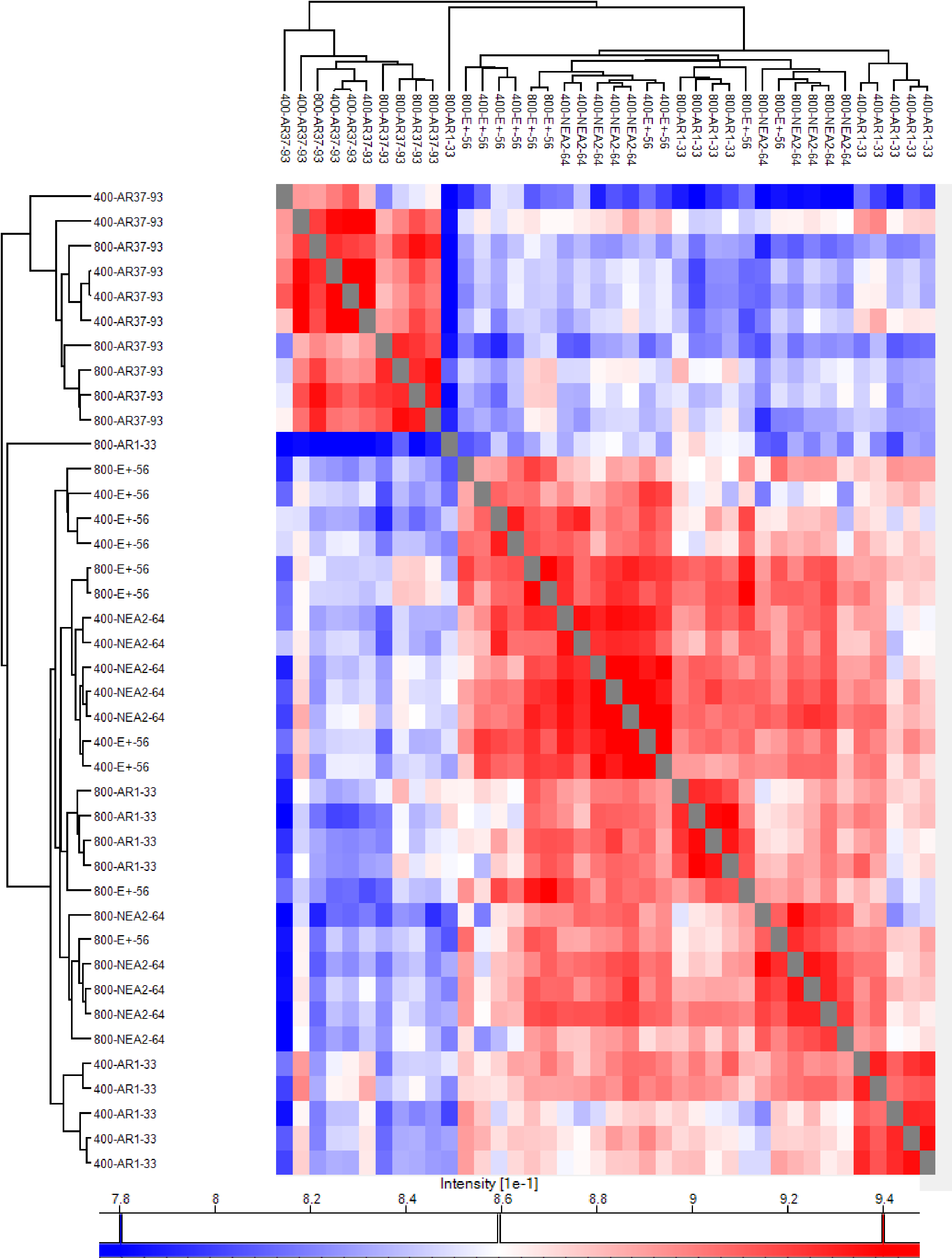

Supplement: Supplementary file 1 [file jof-06-00360-s001.zip › supplemental figure 1.png]
